# Selective Synthesis of Tetrahydroisoquinoline and Piperidine Scaffolds by Oxidative Ring Opening/Ring Closing Protocols of Substituted Indenes and Cyclopentenes

**DOI:** 10.1002/open.202400475

**Published:** 2024-12-27

**Authors:** Anas Semghouli, László Drahos, Jianlin Han, Loránd Kiss, Melinda Nonn

**Affiliations:** ^1^ Institute of Organic Chemistry Stereochemistry Research Group HUN-REN Research Center for Natural Sciences H-1117 Budapest Magyar tudósok krt. 2 Hungary; ^2^ MTA TTK Lendület Artificial Transporter Research Group Institute of Materials and Environmental Chemistry HUN-REN Research Center for Natural Sciences H-1117 Budapest Magyar tudósok krt. 2 Hungary; ^3^ Institute of Organic Chemistry MS Proteomics Research Group HUN-REN Research Centre for Natural Sciences H-1117 Budapest Magyar tudósok krt. 2 Hungary; ^4^ Jiangsu Co-Innovation Center of Efficient Processing and Utilization of Forest Resources College of Chemical Engineering Nanjing Forestry University Nanjing 210037 China; ^5^ National Drug Research and Development Laboratory HUN-REN Research Centre for Natural Sciences Magyar tudósok krt. 2 1117 Budapest Hungary

**Keywords:** Cyclization, Ring expansion, Ring opening, Ring closing, Piperidine, Tetrahydroisoquinoline

## Abstract

Novel tetrahydroisoquinoline and piperidine derivatives were selectively synthesized from substituted indenes or cyclopentenes. The process starts with an oxidative cleavage of the ring olefin bond, which gives reactive diformyl intermediates. By a ring‐closing step using chiral (*R*) or (*S*) α‐methylbenzylamine under a reductive amination protocol facilitated ring formation with ring expansion of the corresponding nitrogen‐containing heterocycles. The stereocontrolled methodology enabled accurate control of the stereochemistry of the final products. Additionally, the synthesized amino acid derivatives possessing an aryl moiety in their structure may be relevant building blocks for foldamer chemistry.

## Graphical abstract

Synthesis under stereocontrol of some novel tetrahydroisoquinoline and functionalized piperidine derivatives from substituted indenes or cyclopentenes is described. The key steps of the transformation are an oxidative ring cleavage across the olefin bond, followed by cyclization under double reductive amination with primary chiral amines through ring expansion.

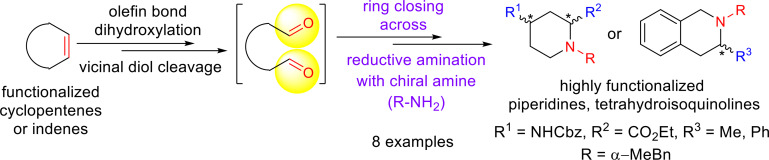

## Introduction

Oxidative cleavage of C=C bonds is one of the paramount reactions used for the preparation of dicarbonyl compounds. This process has gained increasing popularity in organic synthesis. The method is based on the transformation of olefins into 1,2‐diols followed by cleavage of the vicinal diol C−C bond with a suitable oxidant. Common methods employed to perform these operations are the Upjohn method, the Lemieux–von Rudloff oxidation, and the ozonolysis reaction.[^1^, ^2^, ^3^, ^4^, ^5^, ^6^] The Upjohn method is a two‐step process for C=C bond cleavage. The first step is dihydroxylation with the OsO_4_/NMO system. In the second step, NaIO_4_ oxidizes the vicinal *cis*‐diols, yielding dicarbonyl products. Additionally, reaction of the resulting dicarbonyl compound with an amine leads to the formation of a saturated azaheterocycle ring.[^7^, ^8^, ^9^]

Azaheterocyclic derivatives are a crucial and widely prevalent group of compounds in pharmaceutical and medicinal chemistry. Over the past two decades, the chemistry of functionalized azaheterocycles has gained significant importance. These compounds are abundant in nature, and they play a vital role in medicinal chemistry and drug design. Examples include alkaloids, anticancer agents, antibiotics, antiviral agents, vitamins, and numerous drugs, containing a saturated azaheterocyclic ring core. The ring size, along with the nature and the stereochemical characteristics of the substituents, is essential in determining their biological properties.[^10^, ^11^, ^12^, ^13^, ^14^, ^15^]

**Figure 1:**
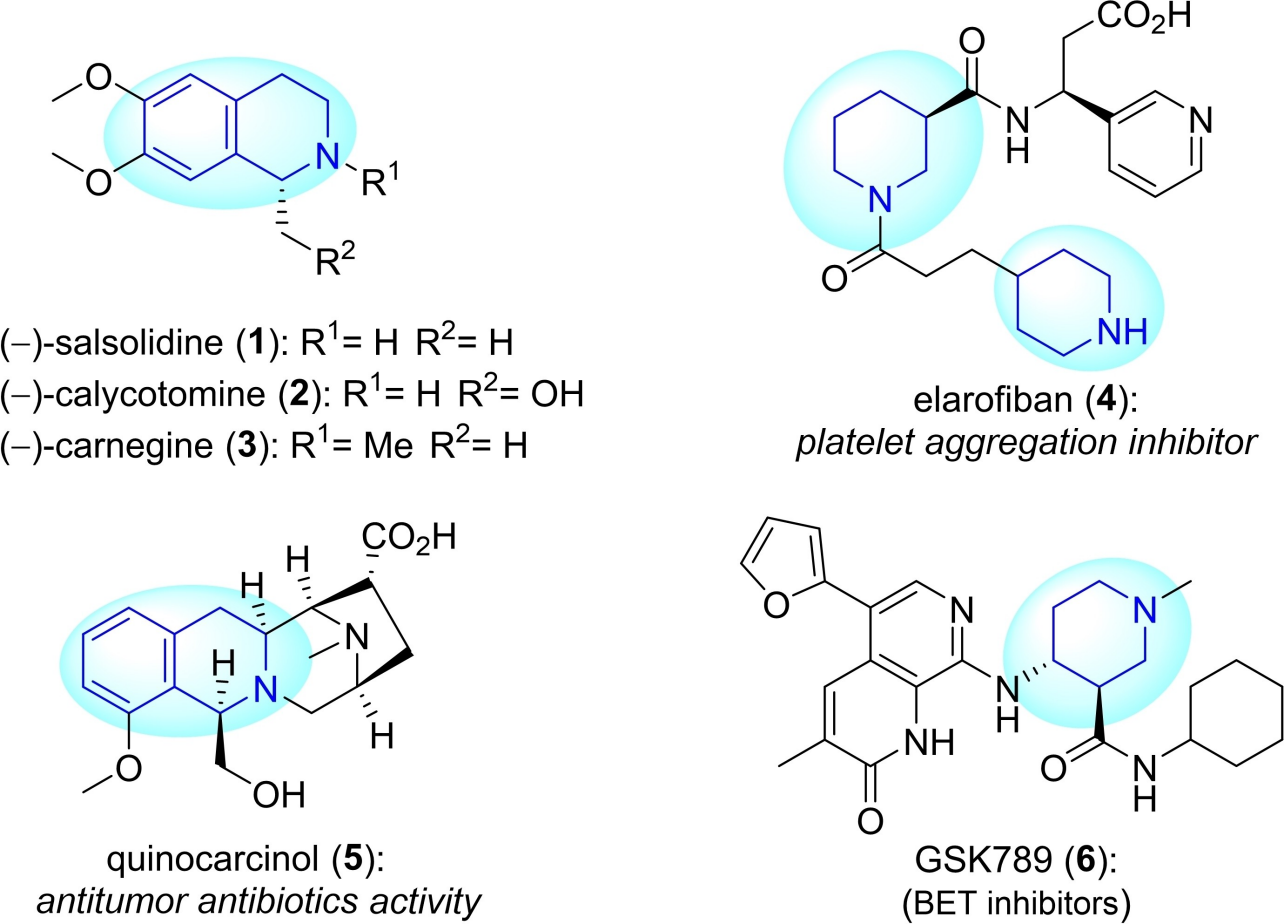
Tetrahydroisoquinoline and piperidine motifs found in natural products and bioactive compounds.

**Figure 2:**
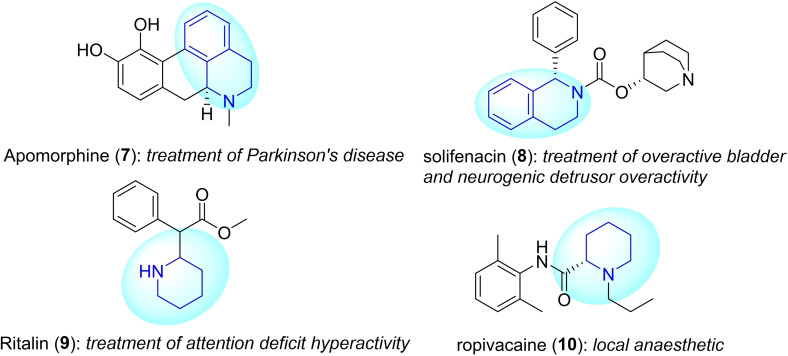
Tetrahydroisoquinoline and piperidine motifs found in some drugs.

Furthermore, tetrahydroisoquinoline and piperidine derivatives are an interesting subgroup of this compound family. Each of these subgroups has its own unique structural features and potential applications. A number of natural products and bioactive compounds, such as (−)‐salsolidine **(1)**, (−)‐calycotomine **(2)**, (−)‐carnegine **(3)**, elarofiban **(4)**, quinocarcinol **(5)**, and GSK789 **(6)**, are depicted on Figure 1. Figure 2 shows some notable drugs like solifenacin **(8)**, used to treat overactive bladder and neurogenic detrusor overactivity, and Ritalin **(9)** applied for treating attention deficit hyperactivity.[^16^, ^17^, ^18^, ^19^, ^20^, ^21^, ^22^] As a consequence, such compounds and their synthesis have always stood out as a highlighted research topic.

**Figure 3:**
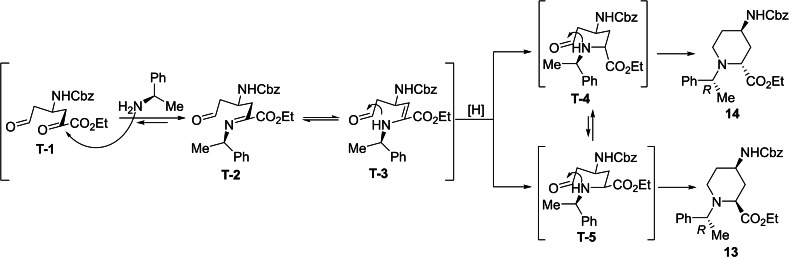
A possible route to the formation of diastereoisomers 13 and 14.

**Scheme 1:**
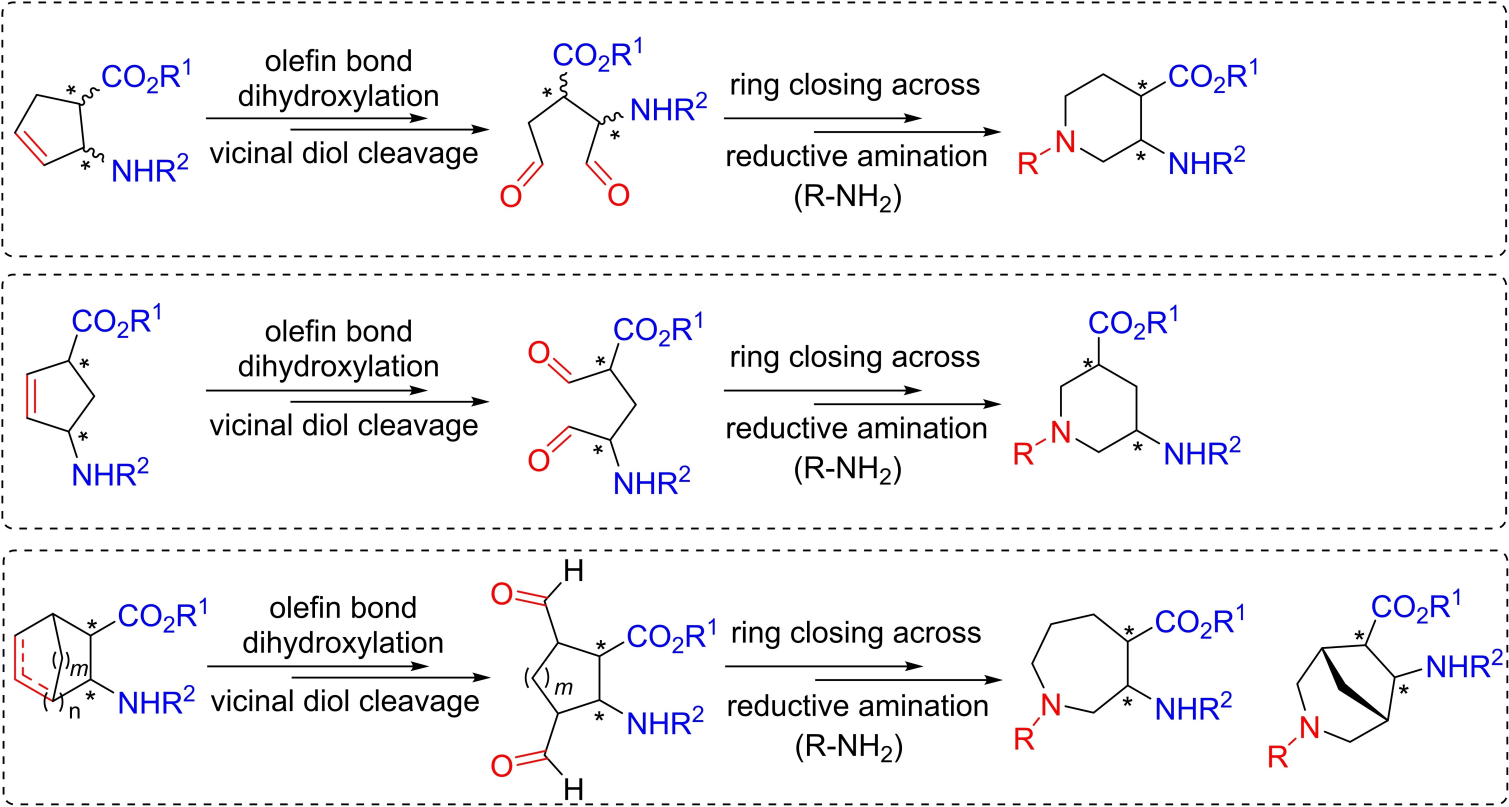
Earlier work: synthesis of diverse azaheterocyclic *β*‐amino acids via dihydroxylation/vicinal diol cleavage/ring closing across reductive amination (*n*=0, 1, 3, *m*=0, 1; R^1^=H, Et, Bn; R^2^=Boc, COPh, Cbz)

Our research group reported earlier a synthetic protocol involving the oxidative ring cleavage of unsaturated cyclic *β*‐aminocarboxylates through the ring C=C bond followed by ring closing by double reductive amination giving the products via ring expansion (Figure 3).[^23^, ^24^, ^25^, ^26^, ^27^, ^28^, ^29^, ^30^] This synthesis approach is particularly noteworthy as it opens new avenues for creating diverse and structurally intricate azaheterocyclic *β*‐amino acids, paving the way for potential applications in pharmaceutical and medicinal chemistry (Scheme 1).

**Scheme 2:**
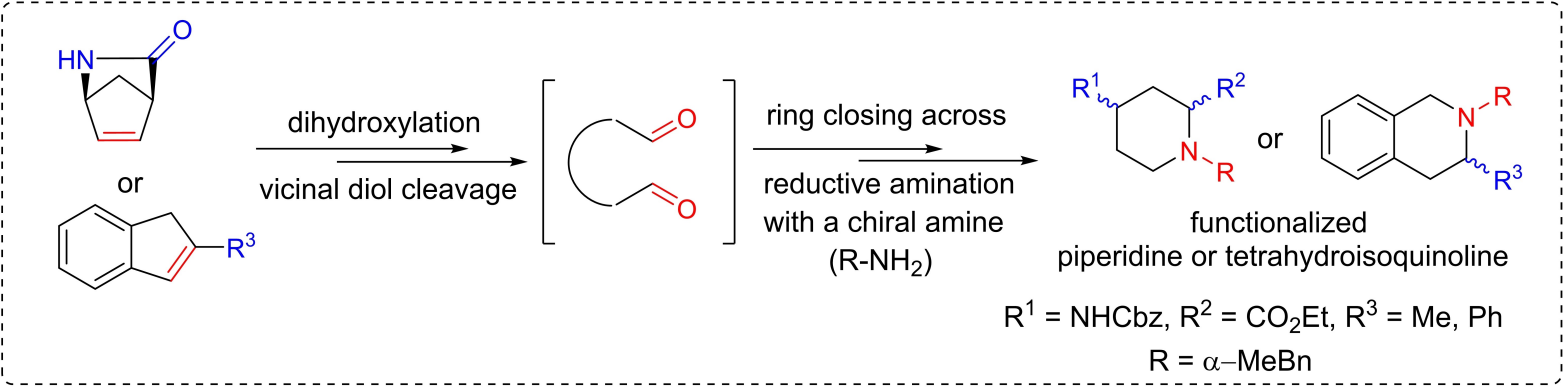
Current work: synthesis of azaheterocycles with tetrahydroisoquinoline and piperidine motifs through dihydroxylation/vicinal diol cleavage/ring closing across reductive amination.

To demonstrate the versatility and reliability of this method, the aim of the current work was to apply olefin bond functionalization, diol formation, oxidative ring cleavage, and ring closure by reductive amination to access azaheterocycles with tetrahydroisoquinoline and piperidine motifs, which are highly valuable building blocks in organic synthesis. The starting compounds for this investigation included alkyl/aryl indenes or a bicyclic *γ*‐lactam, showcasing our commitment to explore diverse structural motifs in the future (Scheme 2).

## Results and Discussion

**Scheme 3:**
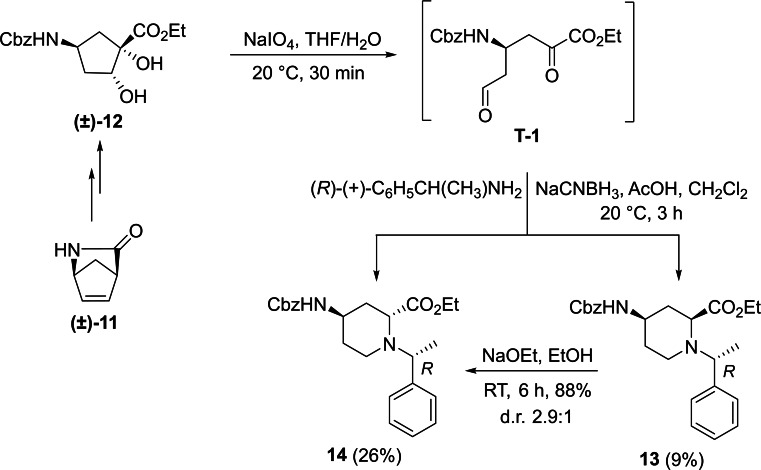
Synthesis of azaheterocyclic *β*‐amino esters 13 and 14 with piperidine motifs.

**Scheme 4:**
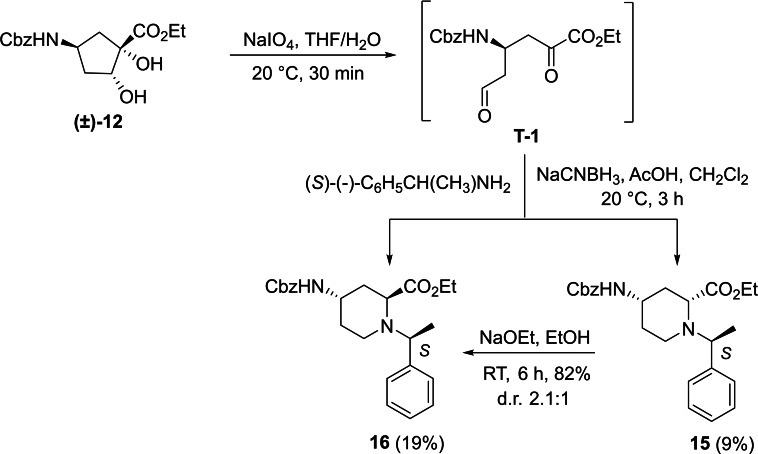
Synthesis of azaheterocyclic *β*‐amino esters 15 and 16 with piperidine motifs.

Racemic Cbz‐protected cis‐*β*‐amino ester **(±)‐12** was synthesized from racemic Vince lactam **(±)‐11** according to a literature method. The initial step involved a ring‐opening reaction with hydrochloric acid in ethanol, followed by the protection of the amino group with benzyl chloroformate. The resulting protected amino ester was then treated with NaOEt, inducing ring double‐bond migration. Subsequently, *cis*‐dihydroxylation conducted in the presence of OsO_4_ and *N*‐methylmorpholine *N*‐oxide (NMO) yielded the corresponding vicinal diol **(±)‐12**.^[23]^ In the next step, NaIO_4_‐mediated oxidative ring cleavage of the vicinal diol produced dicarbonyl intermediate **T‐1**. This dicarbonyl compound was submitted to reductive amination steps with (*R*)‐(+)‐C_6_H_5_CH(CH_3_)NH_2_ (Scheme 3) and (*S*)‐(−)‐C_6_H_5_CH(CH_3_)NH_2_ (Scheme 4), followed by the addition of NaCNBH_3_. The reductive amination took place with ring expansion providing the corresponding piperidine *β*‐amino ester diastereoisomers **13** and **14** as well as **15** and **16** in approximately 1 : 3 and 1 : 2 diastereomeric ratios. Epimerization of esters **13** and **15** with NaOEt in EtOH afforded substrates **14** and **16**, in which the ester and the carbamate group are *trans* to each other (Schemes 3 and 4). The structure of the formed azaheterocyclic amino esters (**13**, **14**, **15**, and **16**) was elucidated and certified by means of NMR analyses. It should be noted that even the overall yield of the cyclization is modest (only 35 %), no by‐products, only unidentifiable polymeric materials were detected in this transformation.

In order to improve the yield of the ring closure a series of optimization conditions were tested such as solvent (THF, PhMe, 1,4‐dioxane, EtOH), temperature (0 °C, room temperature or reflux), time (2 h, 3 h, 6 h) and the best condition was found that indicated on Scheme 3 (CH_2_Cl_2_, room temperature, 3 h).

A possible route for the formation of the azaheterocycles **13** and **14** (and analogously for **15** and **16**) is depicted on Figure 3. The α‐methylbenzylamine initiates and attack to the more reactive carbonyl C‐atom of **T‐1** and leads to imine (enamine) **T‐2** (**T‐3**). During the reductive step across a thermodynamic equilibrium between the more stable **T‐4** and less favored **T**‐**5** the corresponding azaheterocycles **14** (major) and **13** (minor) are formed (Note that isomerization occurs during the process **T‐4** and **T‐5**, due to the basic medium of the rection (by involvement of the active H‐atom connected to carboxylate C‐atom, similar isomerizations to the thermodynamically more stable isomer were found earlier, see references [9a] and [30]).

Our next aim was the simultaneous reductive removal of both the Cbz protecting group and the chiral auxiliary. Unfortunately, all our attempts to remove the α‐methylbenzyl unit under various experimental conditions such as hydrogenolysis (H_2_, Pd/C, solvent) or transfer hydrogenation (Pd/C, solvent, HCO_2_NH_4_ or 1,3‐cyclohexadiene) at various temperatures and pressures failed and gave only unidentifiable mixture of products or unreacted material.

**Scheme 5:**
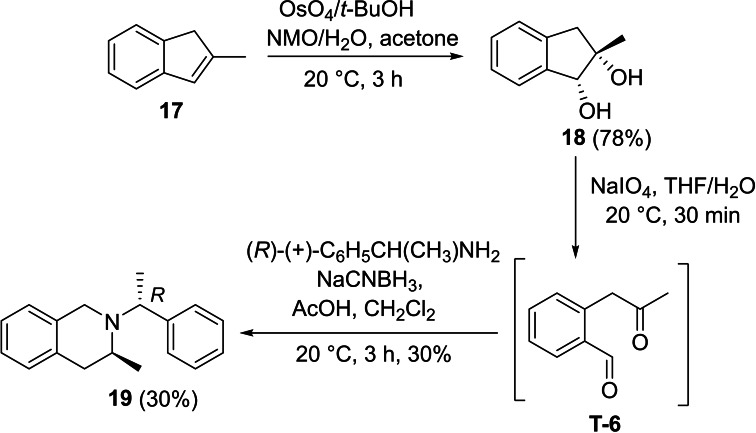
Synthesis of azaheterocyclic product 19 with a tetrahydroisoquinoline motif.

**Scheme 6:**
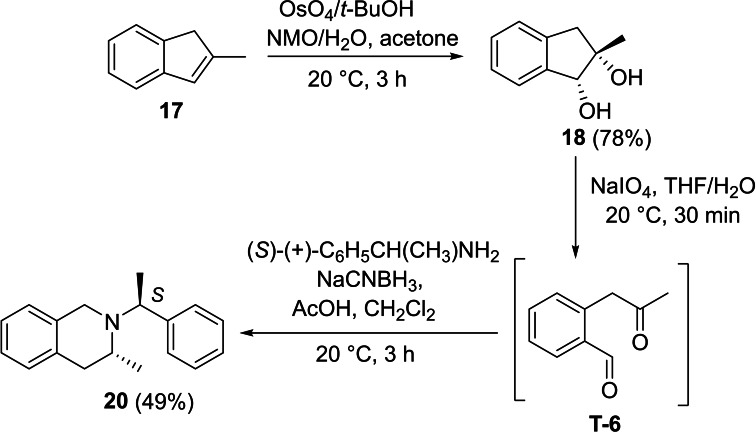
Synthesis of azaheterocyclic product 20 with a tetrahydroisoquinoline motif.

Next, we investigated the applicability of the method for the preparation of tetrahydroisoquinoline scaffolds. First, 2‐methyl‐1*H*‐indene **17** was transformed into the corresponding diol derivatives **18** with NMO/OsO_4_. Then, **18** underwent oxidative ring cleavage with NaIO_4_, followed by ring closure through reductive amination of diformyl intermediate **T‐6** using chiral amines (*R*)‐(+)‐C_6_H_5_CH(CH_3_)NH_2_ (Scheme 5) and (*S*)‐(−)‐C_6_H_5_CH(CH_3_)NH_2_ (Scheme 6). This process, contrary to the cyclization of **T‐1**, yielded the corresponding azaheterocycles **19** and **20** with tetrahydroisoquinoline motifs in a diastereoselective manner (Schemes 5 and 6).

**Scheme 7:**
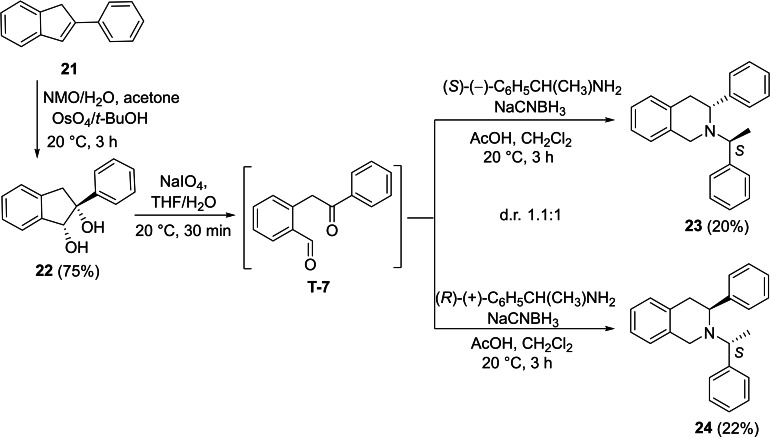
Synthesis of azaheterocycles with tetrahydroisoquinoline motifs 23 and 24.

Further extension of the synthetic methodology for the access of novel structures with tetrahydroisoquinoline core could be performed. Thus, we selected 2‐phenyl‐1*H*‐indene as the starting model compound, which was subjected to oxidation providing the corresponding vicinal diol **22**. NaIO_4_‐mediated oxidative ring opening of this dihydroxylated derivative yielded dicarbonyl intermediate **T‐7**. This intermediate was then subjected to reductive amination with (*R*)‐(+)‐chiral amine and NaBH_3_CN in CH_2_Cl_2_ in the presence of AcOH. Cyclization into the corresponding tetrahydroisoquinoline framework took place affording compound **23** in a 20 % yield. Under the same conditions, but with (*S*)‐(−)‐chiral amine, the expected cyclization occurred, and it gave (*S*)‐3‐phenyl‐2‐((*R*)‐1‐phenylethyl)‐1,2,3,4‐tetrahydroisoquinoline **24** (Scheme 7).

Unfortunately, all our efforts in order to remove the methylbenzyl moiety in **19**, **20**, **23** or **24** under reductive conditions failed again. Under milder conditions unreacted material was isolated, while under forcing hydrogenolysis media, only a polymeric mixture of materials was formed.

## Conclusions

The application of ring olefin bond transformation of some indene or cyclopentene skeleton, serving as model compounds, allowed the generation of diverse tetrahydroisoquinoline frameworks, as well as the synthesis of an azaheterocyclic *β*‐amino esters with piperidine motifs. These procedures involved oxidative ring cleavage followed by ring closure with double reductive amination of dicarbonyl intermediates in the presence of chiral amines :[Q1]  (*R*)‐α‐methylbenzylamine and (*S*)‐α‐methylbenzylamine. The outcome of the ring opening/ring closing procedure was examined under various experimental conditions investigating the substrate influence. It was observed that, under double reductive amination, γ‐aminocylopentenecarboxylic ester derived diformyl intermediate (**T‐1**) afforded the diastereomeric mixture of piperidine scaffolds in 3 : 1 and 2 : 1 ratio. Cyclization of dicarbonyl intermediates **T‐6** and **T‐7** derived from methyl‐ or phenylindene, in turn, provided the corresponding tetrahydroisoquinoline derivatives with full selectivity. To sum up, based on the data reported in the current work and our earlier findings,[^23^, ^24^, ^25^, ^26^, ^27^, ^28^, ^29^, ^30^] it is evident that our synthesis approach proves to be an effective method for the creation of diverse and structurally intricate azaheterocyclic derivatives. These compounds are highly valuable building blocks in organic synthesis, and they might have potential applications in pharmaceutical chemistry. Furthermore, the prepared amino acid derivatives possessing an aryl element in their structure, in view of the influence on the secondary structures of peptides,^[31]^ can be regarded as highly interesting building blocks in foldamer chemistry.

## Experimental Part

### General Information

Chemicals were obtained from Merck. Solvents were used as received from the supplier. Silica gel 60 F254 was bought from Merck. Measurement of melting points was done with a Kofler apparatus. NMR spectra were recorded at room temperature using a Bruker Avance Neo 500 spectrometer with an 11.75 T magnetic field (^1^H frequency 500.20 MHz, ^13^C frequency 125.78 MHz) in CDCl_3_ solution, using deuterium signal of the solvent to lock the field. The chemical shifts of ^1^H and ^13^C are given relative to TMS. High‐resolution mass spectrometry (HRMS) measurements were performed on either a Thermo Scientific Q‐Exactive Plus Orbitrap mass spectrometer (Thermo Fisher Scientific Inc., Budapest, Hungary) equipped with an electrospray ionization ion source in the positive ionization mode, or a Q‐TOF Premier mass spectrometer (Waters Corporation, Milford, MA, USA) in positive electrospray ionization.

Compound (±)‐12 was synthesized according to Ref. [23] (L. Ouchakour, R. A. Ábrahámi, E. Forró, M. Haukka, F. Fülöp, L. Kiss, *Eur. J. Org. Chem*. **2019**, *12*, 2202–2211. doi:10.1002/ejoc.201801540.)

### General Procedure for Dihydroxylation

To a solution of compound containing the C=C double bond (10 mmol) and NMO (1.2 equiv) in acetone (50 mL) was added dropwise to a 2 % OsO_4_ solution in *t*‐BuOH (0.3 mL) and the resulting reaction mixture was stirred for 3 h at room temperature. After the reaction monitored by TLC, termination was performed by adding saturated aqueous of Na_2_SO_3_ (100 mL) and then the mixture was extracted with CH_2_Cl_2_ (3×50 mL). The combined organic phase was dried (Na_2_SO_4_), filtered, and evaporated under reduced pressure. The crude products were purified by means of column chromatography on silica gel (*n*‐hexane/EtOAc).

Note that all utensils (cylinder, syringe etc.) used for handling the osmium derivative (2 % solution in *t*‐BuOH) were neutralized by using three times its volume of vegetable oil.

### General Procedure for the Cleavage of the Olefin Bond of Vicinal Diols

To a stirred solution of dihydroxylated indene (2 mmol) or dihydroxylated azaheterocyclic *β*‐amino ester (2 mmol), NaIO_4_ (1.5 equiv) was added in THF/H_2_O (25 mL/2 mL). After stirring the mixture for 0.5 h at 20 °C under an Ar atmosphere, H_2_O (40 mL) was added. The mixture was then extracted with CH_2_Cl_2_ (3×20 mL) and the combined organic solution was dried over Na_2_SO_4_. The resulting solution containing the dialdehyde derivative was concentrated to half its volume and then it was used without purification for the next reaction step.

### General Procedure for Reductive Amination

To the solution of the dicarbonyl derivative amine ((*R*)‐(+)‐C_6_H_5_CH(CH_3_)NH_2_ (1 equiv) or (*S*)‐(−)‐C_6_H_5_CH(CH_3_)NH_2_) (1 equiv) were added, and the mixture was stirred at 20 °C for 10 min. Next, NaCNBH_3_ (1 equiv) and AcOH (2 drops) were added and stirring was continued for another 3 h at 20 °C. The reaction mixture was diluted with H_2_O (30 mL) and extracted with CH_2_Cl_2_ (3×30 mL). The combined organic phases were dried (Na_2_SO_4_) and concentrated under reduced pressure. The residue was then purified by column chromatography on silica gel (*n*‐hexane/EtOAc).

### General Procedure for Epimerization

To a solution of 0.50 g azaheterocyclic *β*‐amino ester in 15 mL of ethanol (EtOH) 1 equivalent of sodium ethoxide (NaOEt) was added in portions at room temperature. The reaction mixture was stirred for 6 hours and after dilution with water (10 mL) it was extracted with dichloromethane (CH_2_Cl_2_) (3×15 mL). The combined organic layers were dried over sodium sulfate (Na_2_SO_4_), filtered, and concentrated under reduced pressure. The residue was then purified by column chromatography on silica gel.

### Characterization of the Newly Synthesized Compounds




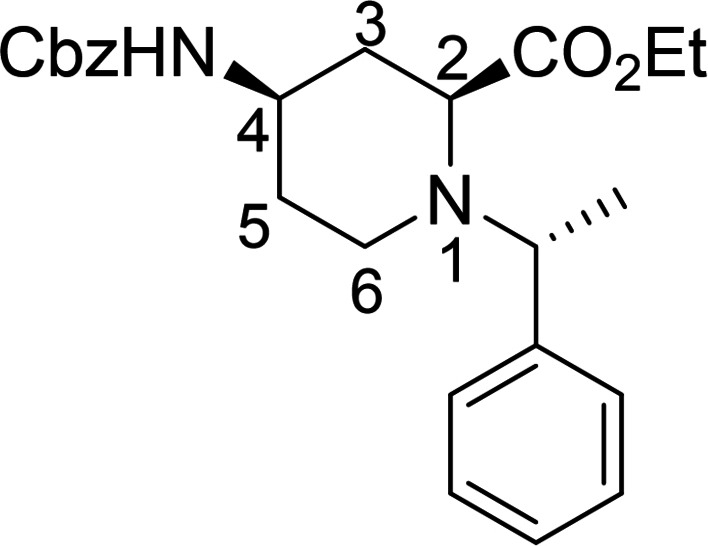




#### Ethyl (2*S*, 4*R*)‐4‐(((benzyloxy)carbonyl)amino)‐1‐((*R*)‐1‐[phenylethyl)piperidine‐2 carboxylate, 13

Prepared from compound (±)‐**12** according to *General procedure for the cleavage of the olefin bond of vicinal diols* then *General procedure for reductive amination* (eluent of column chromatography: *n*‐hexane/acetone 5 : 1). A white solid; Yield: 9 %; *R*_f_=0.28 (*n*‐hexane/Acetone 2 : 1); $\left[\alpha_{D}^{20}\right]$ =+35 (*c*=0.25, EtOH); ^1^H NMR (500 MHz, DMSO): δ=1.23 (t, *J*=7.08 Hz, 3H, CH_3_), 1.33–1.41 (m, 4H, CH_3_, H‐5), 1.61 (q, *J=*23.13 Hz, 1H, H‐5), 1.62–1.76 (m, 2H, H‐3), 1.79–1.86 (m, 1H, H‐6), 2.74–2.84 (m, 1H, H‐6), 3.01–3.12 (m, 2H, H‐2, H‐4), 3.81 (q, *J=*14.02 Hz, 1H, NCH), 4.08–4.24 (m, 2H, CH_2_CH_3_), 4.96 (s, 2H, CH_2_), 7.12–7.20 (m, 3H, CH−Ar), 7.23–7.37 (m, 8H, CH−Ar, NH);

^13^C NMR (125 MHz, DMSO): δ=14.53, 19.13, 31.69, 36.27, 43.39, 47.85, 59.51, 60.69, 63.11, 65.67, 127.55, 127.61, 128.24, 128.28, 128.78,128.84, 137.55, 138.51, 155.70, 173.09 ppm; HRMS calcd. for C_24_H_31_N_2_O_4_^+^ ([M+H]^+^): 411.2284. Found: 411.2283.


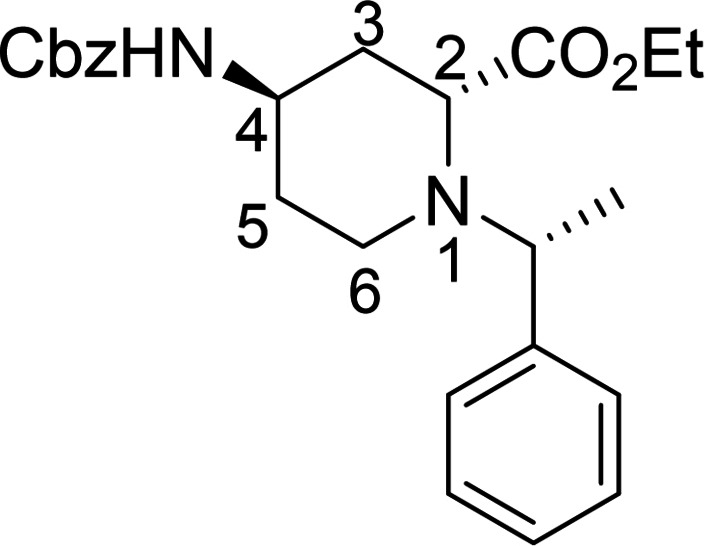




#### Ethyl (2*R*, 4*R*)‐4‐(((benzyloxy)carbonyl)amino)‐1‐((*R*)‐1‐[phenylethyl)piperidine‐2 carboxylate, 14

Prepared from compound (±)‐**12** according to *General procedure for the cleavage of the olefin bond of vicinal diols* then *General procedure for reductive amination* (eluent of column chromatography: *n*‐hexane/acetone 5 : 1). A white solid; Yield: 26 %; *R*_f_=0.32 (*n*‐hexane/Acetone 2 : 1); $\left[\alpha_{D}^{20}\right]$ =+36 (*c*=0.25, EtOH); ^1^H NMR (500 MHz, DMSO): δ=1.17–1.26 (m, 7H, 2CH_3_, H‐3), 1.61 (q, *J=*22.92 Hz, 2H, H‐5), 1.91–1.97 (m, 1H, H‐3), 2.12–2.22 (m, 1H, H‐6), 2.33–2.43 (m, 1H, H‐6), 3.33–3.45 (m, 2H, H‐2, H‐4), 3.85 (q, *J=*13.50 Hz, 1H, NCH), 4.08–4.18 (m, 2H, CH_2_CH_3_), 4.99 (s, 2H, CH_2_), 7.15–7.24 (m, 2H, CH−Ar), 7.28–7.37 (m, 7H, CH−Ar, NH), 7.39–7.45 (m, 2H, CH−Ar).

^13^C NMR (125 MHz, DMSO): δ=10.15, 14.49, 31.70, 36.19, 42.32, 47.84, 57.34, 60.70, 62.28, 65.68, 127.03, 127.18, 127.92, 128.24, 128.29, 128.80, 143.83, 146.62, 155.76, 173.06 ppm.

HRMS calcd. for C_24_H_31_N_2_O_4_^+^ ([M+H]^+^): 411.2284. Found: 411.2287.


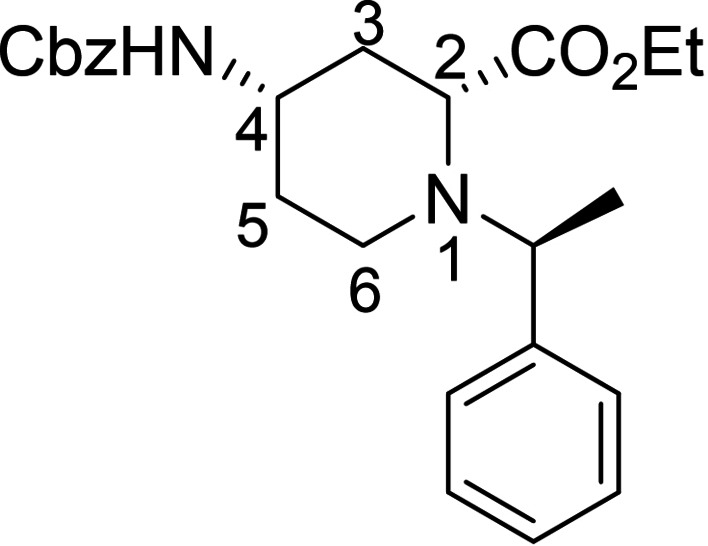




#### Ethyl (2*R*, 4*S*)‐4‐(((benzyloxy)carbonyl)amino)‐1‐((*S*)‐1‐Phenylethyl)piperidine‐2‐Carboxylate, 15

Prepared from compound (±)‐**12** according to *General procedure for the cleavage of the olefin bond of vicinal diols* then *General procedure for reductive amination* (eluent of column chromatography: *n*‐hexane/acetone 5 : 1). A white solid; Yield: Yield: 9 % *R*_f_=0.31 (*n*‐hexane/Acetone 2 : 1); $\left[\alpha_{D}^{20}\right]$ =−31 (*c*=0.25, EtOH); ^1^H NMR (500 MHz, DMSO): δ=1.21–1.26 (t, *J=*7.06 Hz, 3H, CH_3_), 1.34–1.40 (m, 4H, CH_3_, H‐5), 1.43–1.53 (q, *J=*23.22 Hz,1H, H‐5), 1.59–1.73 (m, 2H, H‐3), 1.77–1.85 (m, 1H, H‐6), 2.73–2.81 (m, 1H, H‐6), 2.99–3.11 (m, 2H, H‐4, H‐2), 3.76–3.84(q, *J=*13.86 Hz, 1H, NCH), 4.11–4.22 (m, 2H, CH_2_CH_3_), 4.95 (s, 2H, CH_2_), 7.10–7.18 (m, 2H, CH−Ar), 7.21–7.38(m, 9H, CH−Ar, NH); ^13^C NMR (125 MHz, DMSO): δ=14.55, 19.10, 31.66, 36.27, 43.34, 47.82, 59.46, 60.73, 63.13, 65.67, 127.53, 127.64, 128.28, 128.33, 128.81, 128.88, 137.50, 138.31, 155.69, 173.09 ppm; HRMS calcd. for C_24_H_31_N_2_O_4_^+^ ([M+H]^+^): 411.2284. Found: 411.2285.


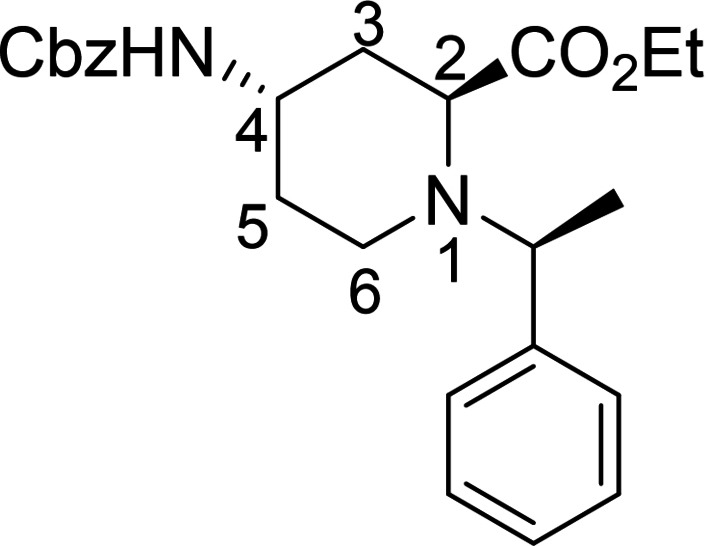




#### Ethyl (2*S*, 4*S*)‐4‐(((benzyloxy)carbonyl)amino)‐1‐((S*)‐1‐Phenylethyl)piperidine‐2‐Carboxylate, 16

Prepared from compound (±)‐**12** according to *General procedure for the cleavage of the olefin bond of vicinal diols* then *General procedure for reductive amination* (eluent of column chromatography: *n*‐hexane/acetone 5 : 1). A white solid; Yield: 19 % *R*_f_=0.32 (*n*‐hexane/Acetone 2 : 1); $\left[\alpha_{D}^{20}\right]$ =−23 (*c*=0.25, EtOH); ^1^H NMR (500 MHz, DMSO): δ=1.17–1.26 (m, 7H, 2CH_3_, H‐5), 1.54–1.73 (m, 2H, H‐5, H‐3), 2.04–2.12 (m, 1H, H‐3), 2.33–2.43 (m, 1H, H‐6), 2.69–2.79 (m, 1H, H‐6), 3.40–3.49 (m, 1H, H‐2), 3.79–3.90 (q, *J=*13.19 Hz, 1H, NCH), 3.94–4.02 (m, 1H, H‐4), 4.08–4.19 (m, 2H, CH_2_CH_3_), 4.98 (s, 2H, CH_2_), 7.17–7.45 (m, 11H, CH−Ar, NH); ^13^C NMR (125 MHz, DMSO): δ=14.70, 21.63, 32.09, 34.61, 44.59, 45.53, 56.70, 60.22, 61.28, 65.64, 127.17, 127.93, 128.27, 128.33, 128.79, 128.82, 143.80, 146.65, 155.74, 173.10 ppm; HRMS calcd. for C_24_H_31_N_2_O_4_^+^ ([M+H]^+^): 411.2284. Found: 411.2278.


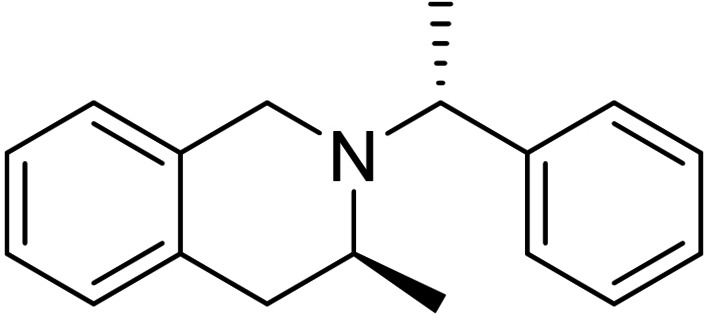




#### (*S*)‐3‐Methyl‐2‐((*R*)‐1‐Phenylethyl)‐1,2,3,4‐Tetrahydroisoquinoline, 19

Prepared from compound **18** according to *General procedure for the cleavage of the olefin bond of vicinal diols* then *General procedure for reductive amination* (eluent of column chromatography: *n*‐hexane/ethyl acetate 6 : 1). Colorless oil; yield: 30 %; *R*_f_=0.34 (*n*‐hexane/EtOAc 4 : 1); $\left[\alpha_{D}^{20}\right]$ =+42 (*c*=0.25, EtOH); ^1^H NMR (500 MHz, CDCl_3_): δ=1.05 (d, J=6.48 Hz, 3H, CH_3_), 1.37 (d, J=6.48 Hz, 3H, CH_3_), 2.51–2.63 (m, 1H, H‐4), 3.10–3.21 (m, 1H, H‐4), 3.43–3.55 (m, 2H, CH_2_), 357–3.66 (m, 1H, H‐3), 3.71–3.79 (q, J=6.47 Hz, 1 H, NCH), 6.84–7.44 (m, 9 H, Ar−H); ^13^C NMR (126 MHz, CDCl_3_): δ=12.84, 19.41, 36.16, 47.52, 47.73, 60.27, 125.31, 125.87, 126.28, 126.74, 127.44, 128.33, 128.47, 129.06, 129.46, 133.81, 134.92, 145.97 ppm; HRMS calcd. for C_18_H_22_N^+^ ([M+H]^+^): 252.1752. Found: 252.1751.


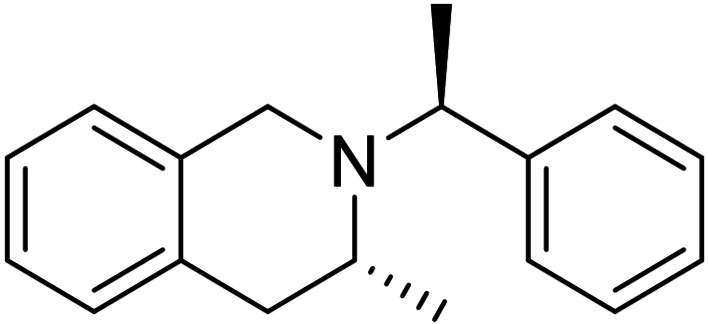




#### (*R*)‐3‐Methyl‐2‐((*S*)‐1‐Phenylethyl)‐1,2,3,4‐Tetrahydroisoquinoline, 20

Prepared from compound **18** according to *General procedure for the cleavage of the olefin bond of vicinal diols* then *General procedure for reductive amination* (eluent of column chromatography: *n*‐hexane/ethyl acetate 6 : 1). Colorless oil; yield: 49 %; *R*_f_=0.35 (*n*‐hexane/EtOAc 4 : 1); $\left[\alpha_{D}^{20}\right]$ =−41 (*c*=0.25, EtOH); ^1^H NMR (500 MHz, CDCl_3_): δ=1.04 (d, *J*=6.62 Hz, 3H, CH_3_), 1.37 (d, *J*=6.67 Hz, 3H, CH_3_), 2.54–2.60 (m, 1H, H‐4), 3.13–3.19 (m, 1H, H‐4), 3.45–3.54 (m, 2H, CH_2_), 358–3.64 (m, 1H, H‐3), 3.74 (q, *J*=6.63 Hz, 1 H, NCH), 6.83–7.43 (m, 9 H, Ar−H); ^13^C NMR (126 MHz, CDCl_3_): δ: 12.89, 19.39, 36.17, 47.56, 47.73, 60.27, 125.32, 125.87, 126.28, 126.74, 127.25, 127.44, 128.33, 128.47, 129.05, 133.81, 134.92, 145.78 ppm; HRMS calcd. for C_18_H_22_N^+^ ([M+H]^+^): 252.1752. Found: 252.1756.


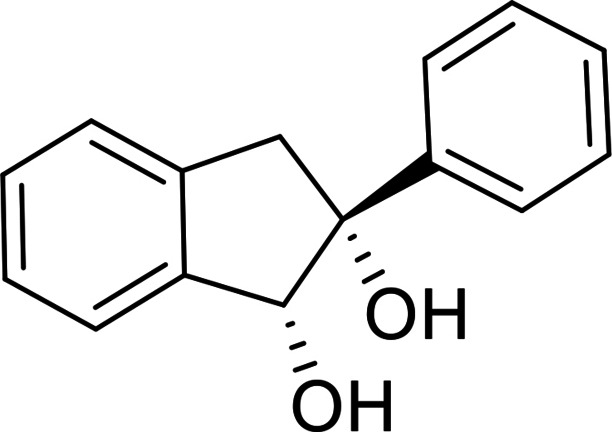




#### (1*R*, 2*R*)‐2‐Phenyl‐2,3‐Dihydro‐1H‐Indene‐1,2‐Diol, 22

Prepared from compound **21** according to *General procedure for dihydroxylation* (eluent of column chromatography: *n*‐hexane/acetone 4 : 1). A white solid; Yield: 75 % *R*_f_=0.38 (*n*‐hexane/Acetone 2 : 1); $\left[\alpha_{D}^{20}\right]$ =+32 (*c*=0.25, EtOH); ^1^H NMR (500 MHz, CDCl_3_): δ=2.57 (d, J=6.41 Hz, 1H, CH_2_), 2.93 (s, 1H, CH), 3.35 (d, J=6.41 Hz, 1H, CH_2_), 3.42 (d, J=7.24 Hz, 1H, OH), 5.34 (d, J=7.32 Hz, 1H, OH), 7.24–7.66 (m, 9 H, Ar−H); ^13^C NMR (126 MHz, CDCl_3_): δ: 47.52, 87.42, 93.27, 125.34, 125.83, 126.28, 126.44, 126.74, 127.33, 128.06, 128.46, 128.83, 143.28, 143.46, 145.98 ppm; HRMS calcd. for C_15_H_14_O_2_Na^+^ ([M+Na]^+^): 249.0891. Found: 249.0891.


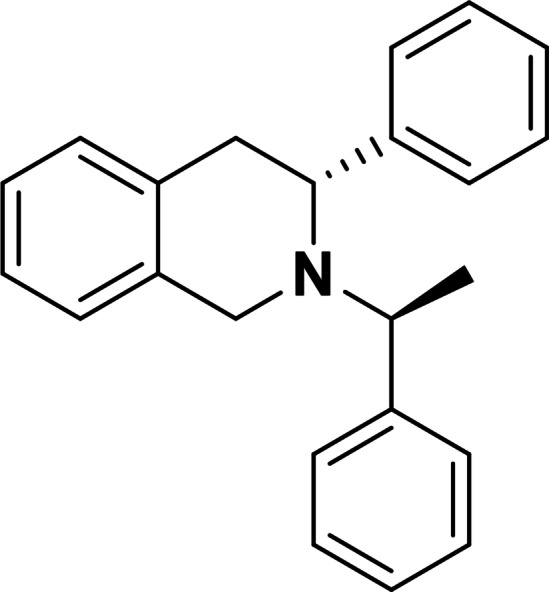




#### (*R*)‐3‐Phenyl‐2‐((*S*)‐1‐Phenylethyl)‐1,2,3,4‐Tetrahydroisoquinoline, 23

Prepared from compound **22** according to *General procedure for the cleavage of the olefin bond of vicinal diols* then *General procedure for reductive amination* (eluent of column chromatography: *n*‐hexane/acetone 6 : 1). Colorless oil; yield: 20 %; *R*_f_=0.44 (*n*‐hexane/acetone 4 : 1); $\left[\alpha_{D}^{20}\right]$ =−26 (*c*=0.25, EtOH); ^1^H NMR (500 MHz, CDCl_3_): δ=1.29 (d, *J*=6.73 Hz, 3H, CH_3_), 3.03–3.21(m, 2H, H‐4), 3.52–3.59 (m, 1H, H‐1), 3.74–3.81(m, 1H, H‐1), 3.95–4.06 (m, 2H, NCH, H‐3), 6.88–7.50 (m, 14H, CH−Ar); ^13^C NMR (125 MHz, CDCl_3_): δ=11.76, 37.32, 46.57, 56.24, 60.79, 125.65, 126.01, 126.31, 126.55, 127.24, 127.60, 128.01, 128.02, 128.11, 128.54, 134.64, 135.48, 143.32, 144.36 ppm; HRMS calcd. for C_23_H_24_N^+^ ([M+H]^+^): 314.1909. Found: 314.1913.


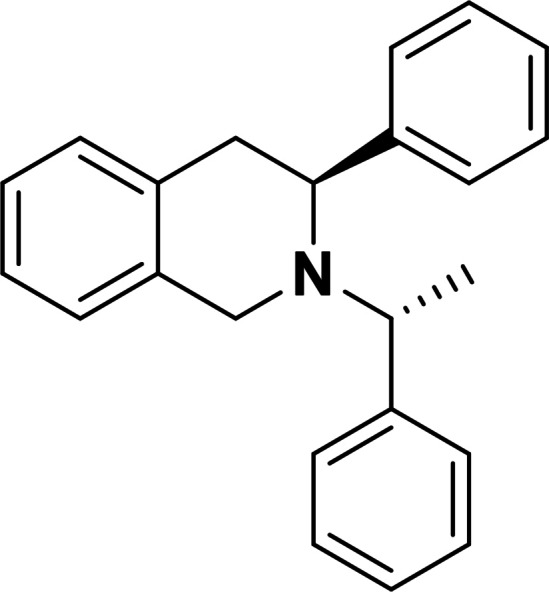




#### (*S*)‐3‐Phenyl‐2‐((*R*)‐1‐Phenylethyl)‐1,2,3,4‐Tetrahydroisoquinoline, 24

Prepared from compound **22** according to *General procedure for the cleavage of the olefin bond of vicinal diols* then *General procedure for reductive amination* (eluent of column chromatography: *n*‐hexane/acetone 6 : 1). Colorless oil; yield: 22 %; *R*_f_=0.44 (*n*‐hexane/Acetone 4 : 1); $\left[\alpha_{D}^{20}\right]$ =+42 (*c*=0.25, EtOH; ^1^H NMR (500 MHz, CDCl_3_): δ=1.29 (d, *J*=6.73 Hz, 3H, CH_3_), 3.03–3.22(m, 2H, H‐4), 3.52–3.61 (m, 1H, H‐1), 3.73–3.82(m, 1H, H‐1), 3.94–4.07 (m, 2H, NCH, H‐3), 6.90–7.49 (m, 14H, CH−Ar); ^13^C NMR (125 MHz, CDCl_3_): δ=11.77, 37.32, 46.58, 56.24, 60.79, 125.65, 126.01, 126.31, 126.55, 127.24, 127.60, 128.01, 128.02, 128.11, 128.54, 134.64, 135.48, 143.31, 144.36 ppm; HRMS calcd. for C_23_H_24_N^+^ ([M+H]^+^): 314.1909. Found: 314.1910.

## Conflict of Interests

The authors declare no conflict of interest.

1

## Supporting information

As a service to our authors and readers, this journal provides supporting information supplied by the authors. Such materials are peer reviewed and may be re‐organized for online delivery, but are not copy‐edited or typeset. Technical support issues arising from supporting information (other than missing files) should be addressed to the authors.

Supporting Information

## Data Availability

The data that support the findings of this study are available on request from the corresponding author. The data are not publicly available due to privacy or ethical restrictions.
