# Supplementary material for: Selective Synthesis of Tetrahydroisoquinoline and Piperidine Scaffolds by Oxidative Ring Opening/Ring Closing Protocols of Substituted Indenes and Cyclopentenes
Source: ChemistryOpen. 2024 Dec 27;14(5):e202400475. doi: 10.1002/open.202400475 (PMC12075104; doi:10.1002/open.202400475)
Supplement: Supplementary file 1 — Supporting Information [file OPEN-14-e202400475-s001.pdf]

ChemistryOpen

Supporting Information

Selective Synthesis of Tetrahydroisoquinoline and Piperidine Scaffolds by Oxidative Ring Opening/Ring Closing Protocols of Substituted Indenes and Cyclopentenes

Anas Semghouli, László Drahos, Jianlin Han, Loránd Kiss,* and Melinda Nonn*

SUPPORTING INFORMATION

Selective synthesis of tetrahydroisoquinoline and piperidine scaffolds by oxidative ring opening/ring closing protocols of substituted indene and cyclopentenes

Anas Semghouli,[1] **László Drahos,**[3] **Jianlin Han,**[4] **Loránd Kiss,**[1*] **Melinda Nonn**[2*]

[1]*Institute of Organic Chemistry, Stereochemistry Research Group, HUN-REN Research Center for Natural Sciences, H-1117 Budapest, Magyar tudósok krt. 2, Hungary*

[2]*MTA TTK Lendület Artificial Transporter Research Group, Institute of Materials and Environmental Chemistry, HUN-REN Research Center for Natural Sciences, H-1117 Budapest, Magyar tudósok krt. 2, Hungary*

[3]*Institute of Organic Chemistry, MS Proteomics Research Group, HUN-REN Research Centre for Natural Sciences, H-1117 Budapest, Magyar tudósok krt. 2, Hungary*

[4]*Jiangsu Co-Innovation Center of Efficient Processing and Utilization of Forest Resources, College of Chemical Engineering, Nanjing Forestry University, Nanjing 210037, China*

E-mail: nonn.melinda@ttk.hu

kiss.lorand@ttk.hu; kiss.lorand00@gmail.com

Tel: +36-30-1600354

NMR spectra of the newly synthesized compounds

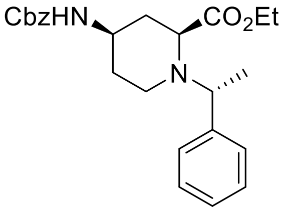

Ethyl (2*S*, 4*R*)-4-(((benzyloxy)carbonyl)amino)-1-((*R*)-1-[phenylethyl)piperidine-2 carboxylate, 13

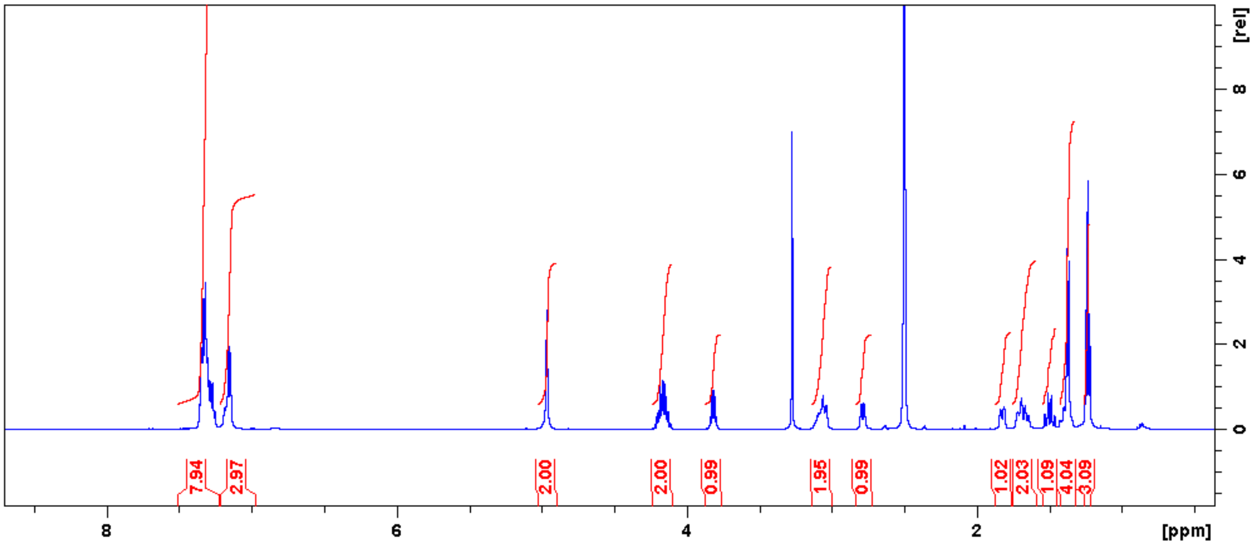

1

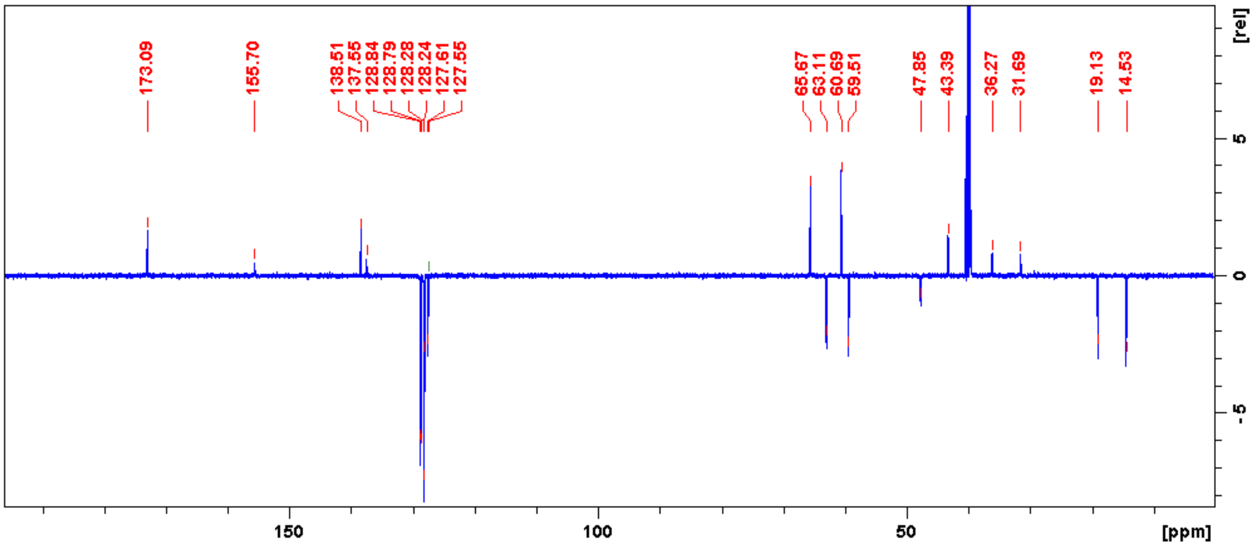

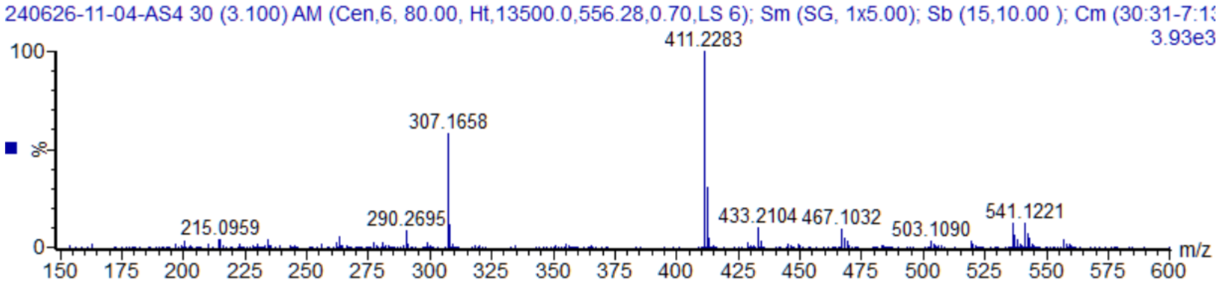

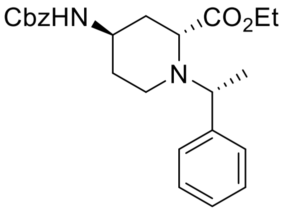

Ethyl (2*R*, 4*R*)-4-(((benzyloxy)carbonyl)amino)-1-((*R*)-1-[phenylethyl)piperidine-2 carboxylate, 14

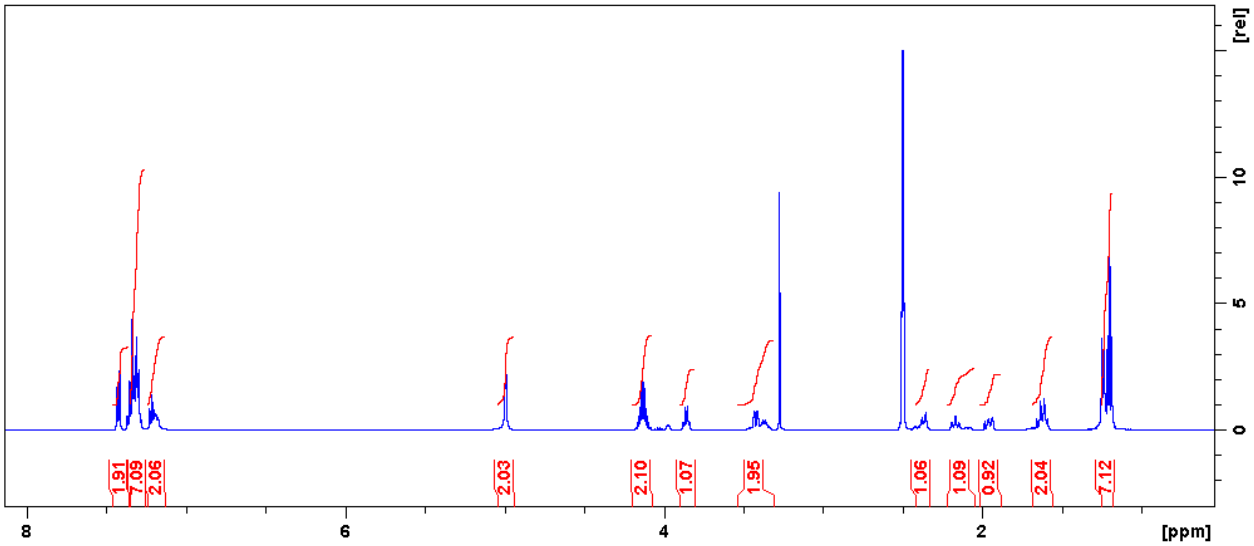

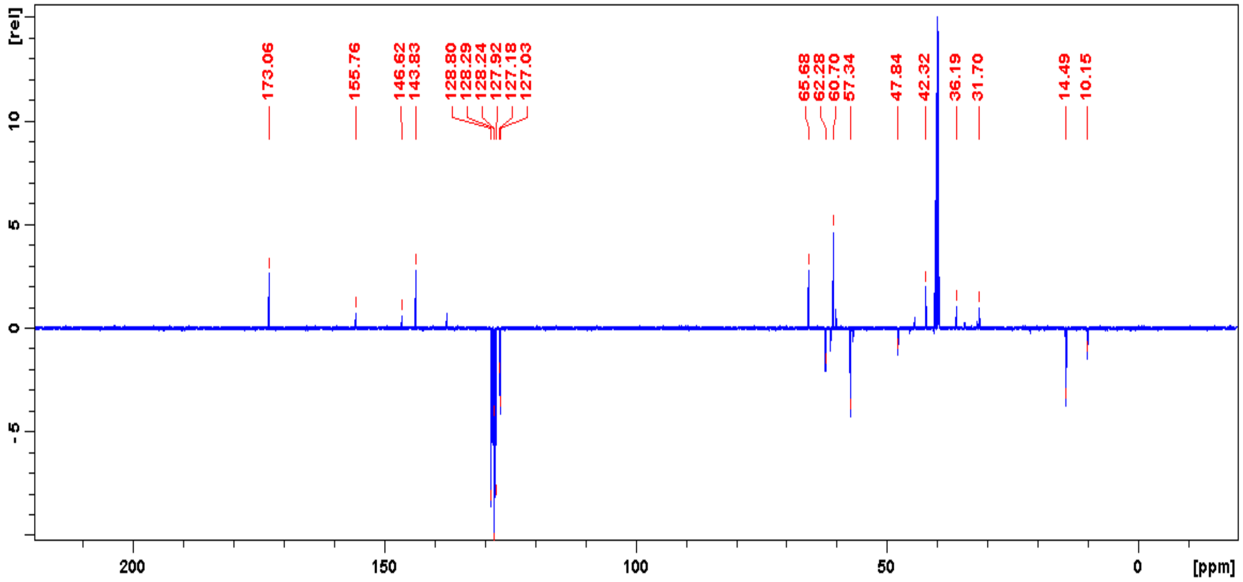

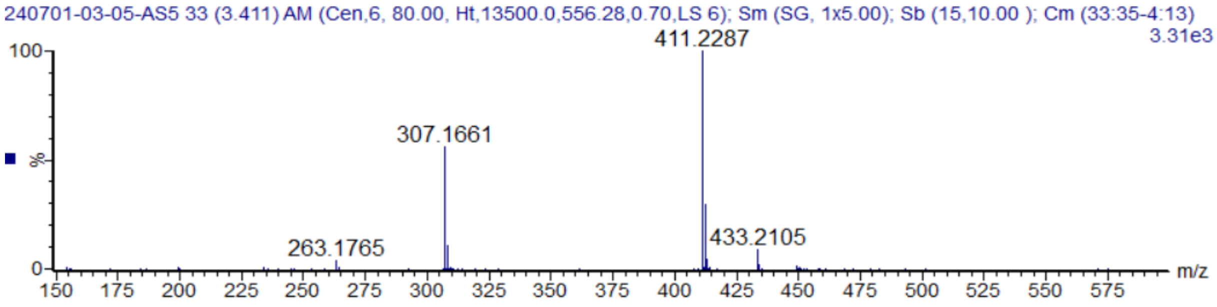

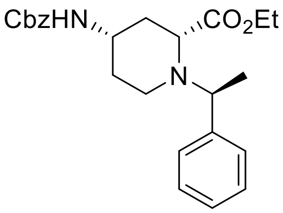

Ethyl (2*R*, 4*S*)-4-(((benzyloxy)carbonyl)amino)-1-((*S*)-1-phenylethyl)piperidine-2-carboxylate, 15

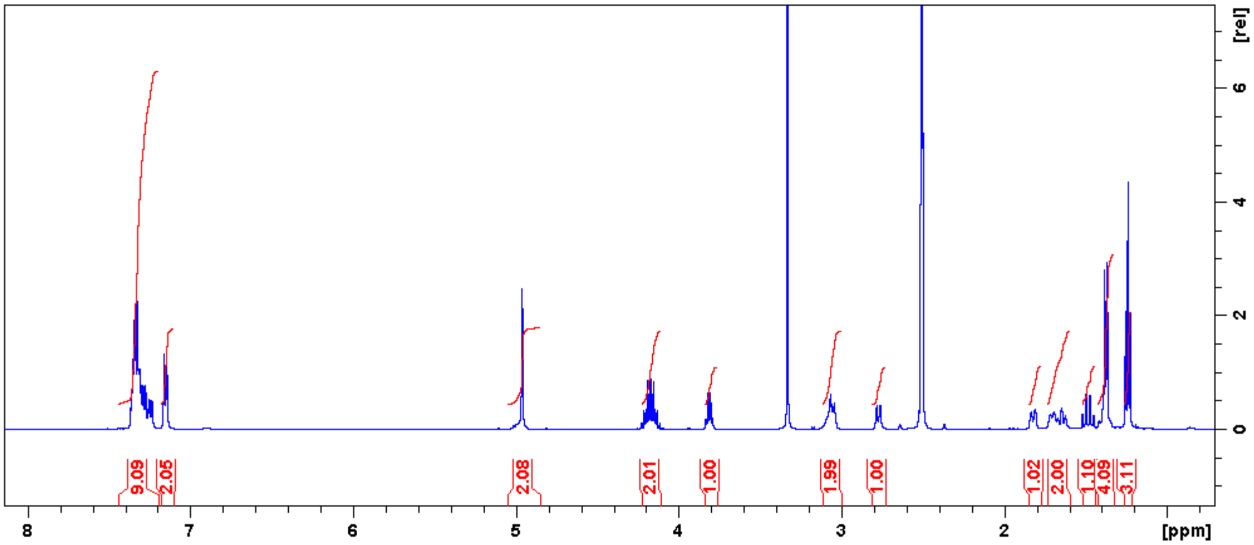
9.09
2.05
2.08
2.01
1.00
1.99
1.00
1.02
2.00
1.10
4.09
3.11
[ppm]
[rel]

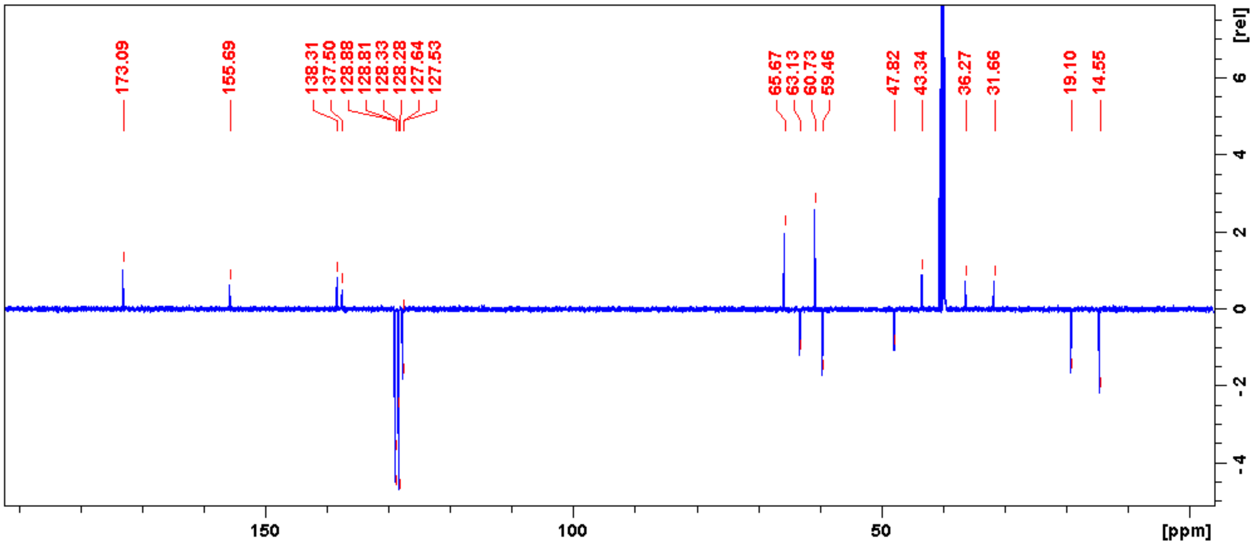
173.09
155.69
138.31
137.50
128.88
128.81
128.33
128.28
127.64
127.53
65.67
63.13
60.73
59.46
47.82
43.34
36.27
31.66
19.10
14.55
[ppm]
[rel]

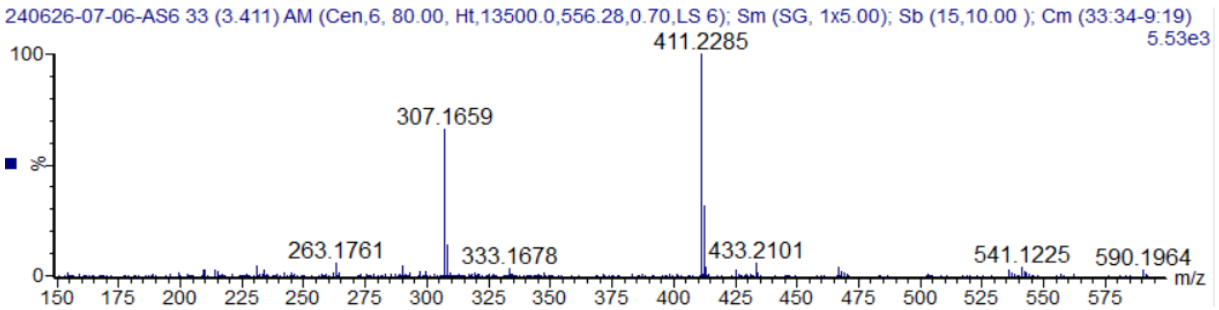
240626-07-06-AS6 33 (3.411) AM (Cen,6, 80.00, Ht,13500.0,556.28,0.70,LS 6); Sm (SG, 1x5.00); Sb (15,10.00); Cm (33:34-9:19)
5.53e3
411.2285
307.1659
263.1761
333.1678
433.2101
541.1225
590.1964
%
m/z

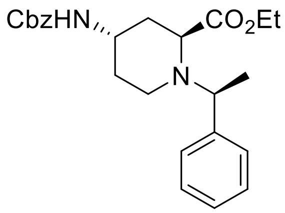

Ethyl (2*S*, 4*S*)-4-(((benzyloxy)carbonyl)amino)-1-((*S*)-1-phenylethyl)piperidine-2-carboxylate, 16

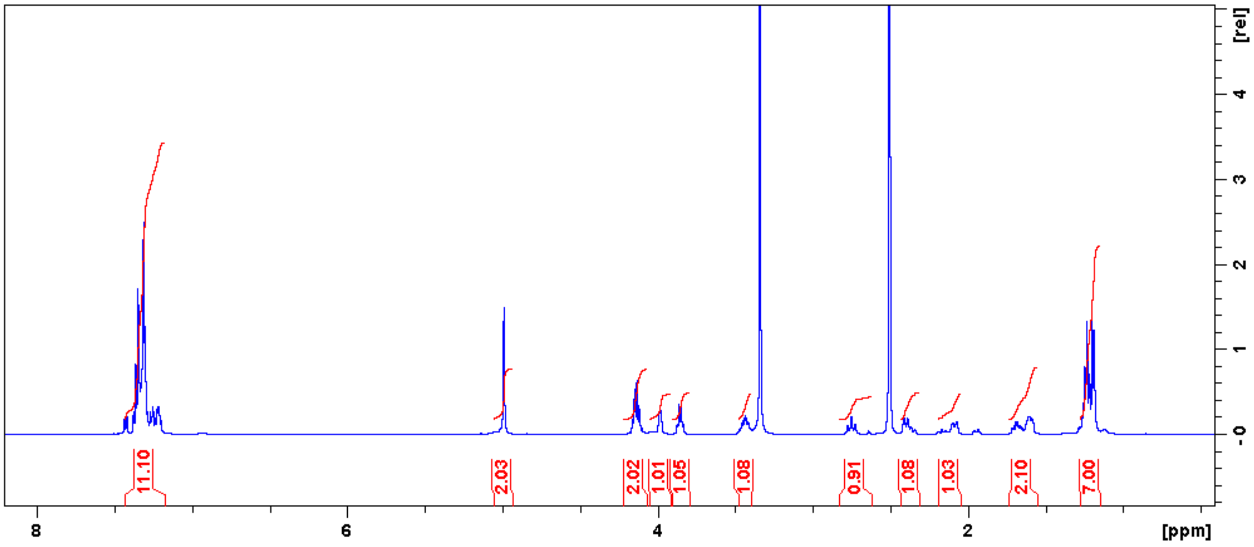

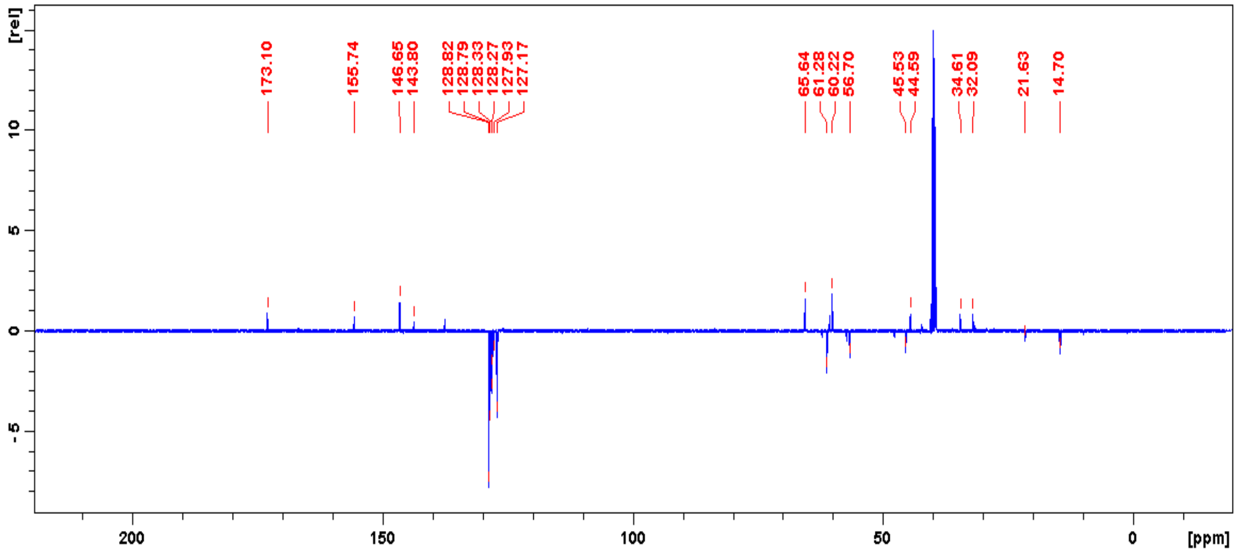

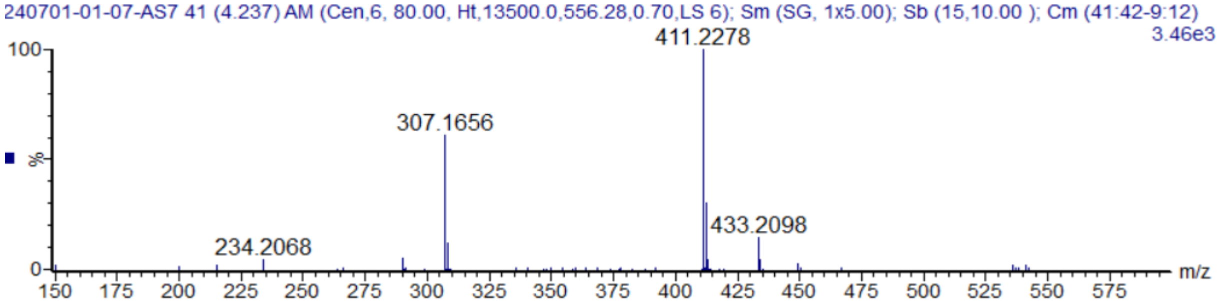

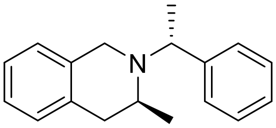

(*S*)-3-methyl-2-((*R*)-1-phenylethyl)-1,2,3,4-tetrahydroisoquinoline, 19

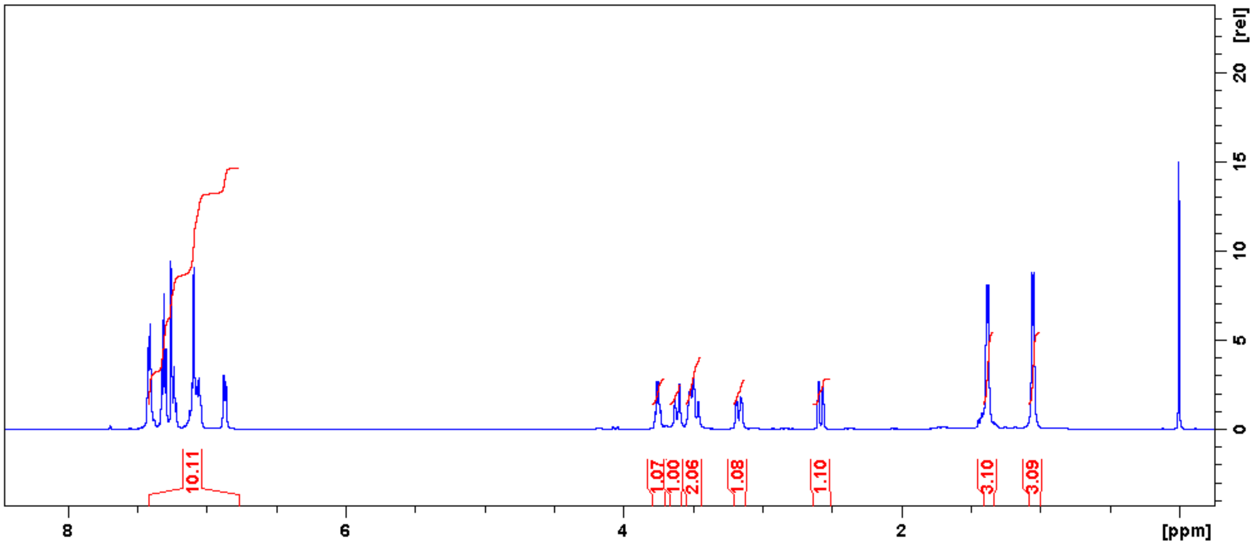

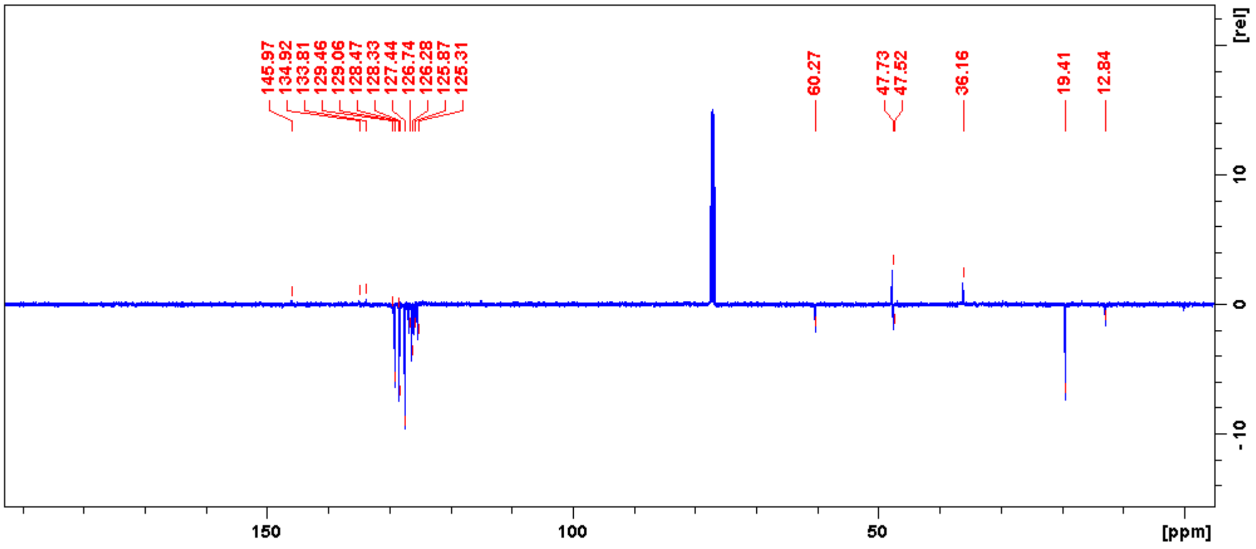

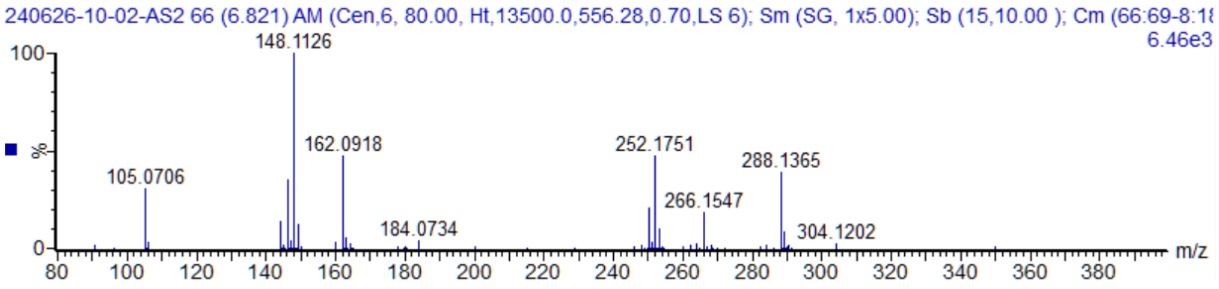

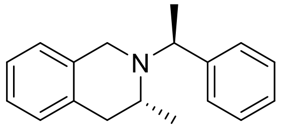

(*R*)-3-methyl-2-((*S*)-1-phenylethyl)-1,2,3,4-tetrahydroisoquinoline, 20

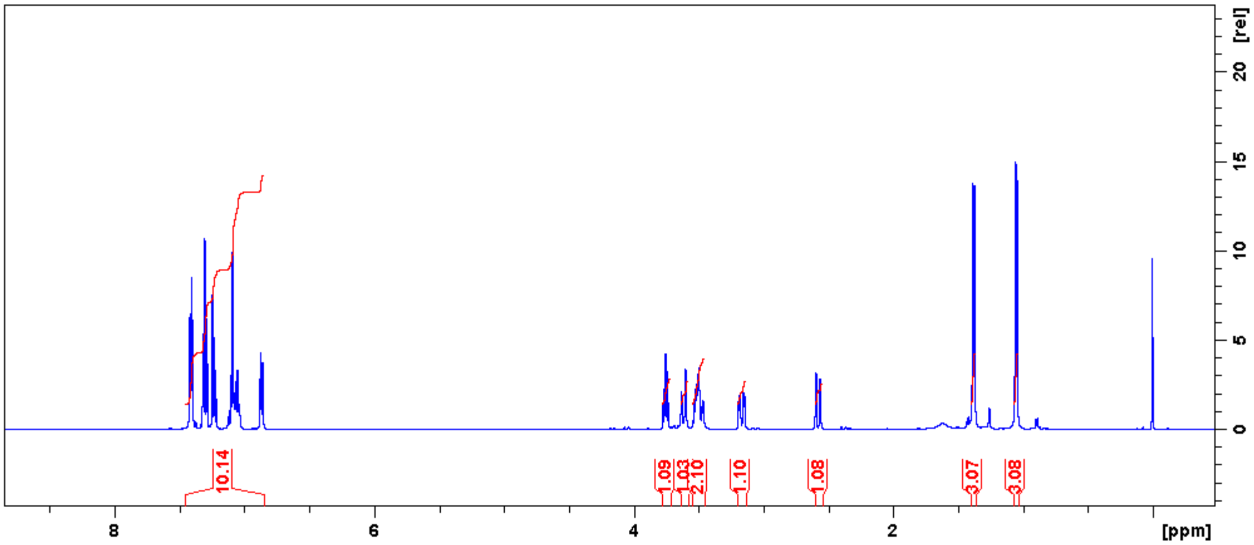
10.14
1.09
1.03
2.10
1.10
1.08
3.07
3.08
[rel]
[ppm]

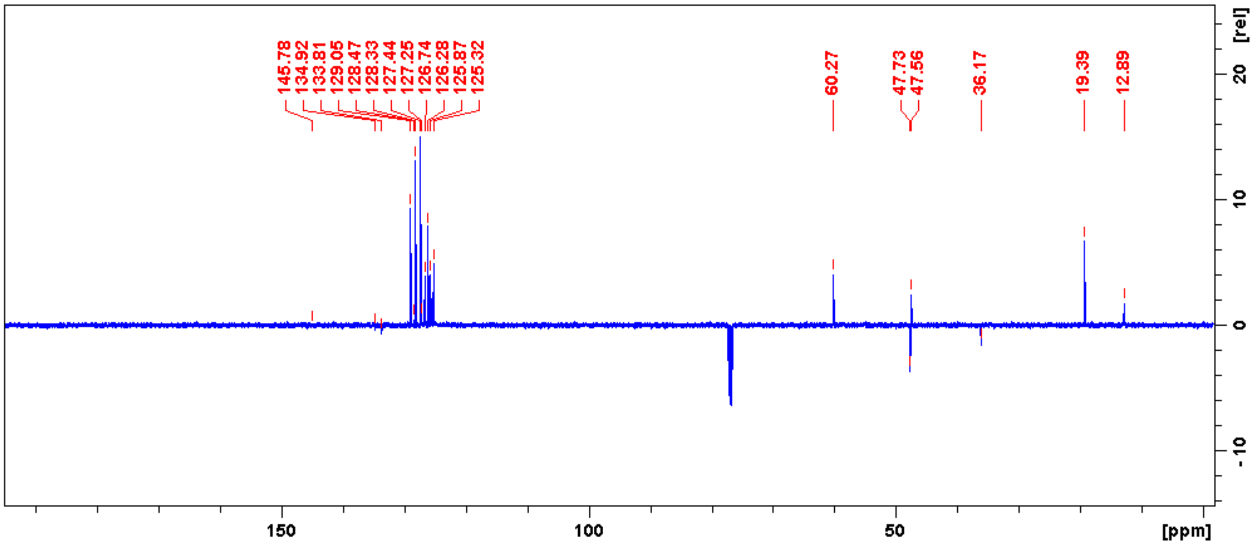
145.78
134.92
133.81
129.05
128.47
128.33
127.44
127.25
126.74
126.28
125.87
125.32
60.27
47.73
47.56
36.17
19.39
12.89
[rel]
[ppm]

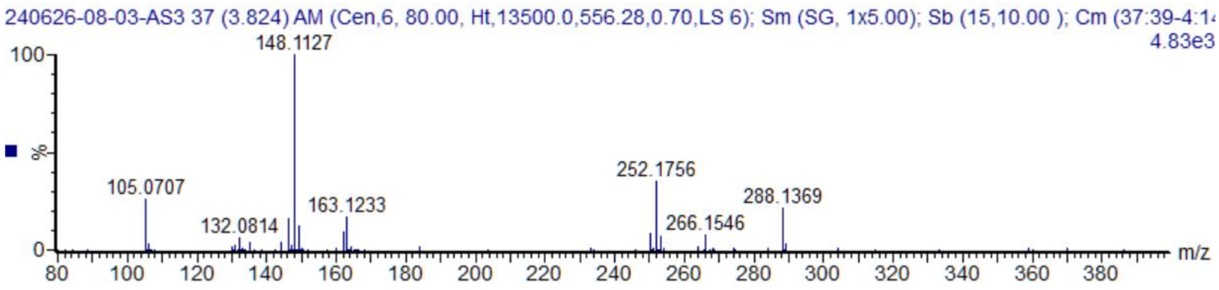
240626-08-03-AS3 37 (3.824) AM (Cen,6, 80.00, Ht,13500.0,556.28,0.70,LS 6); Sm (SG, 1x5.00); Sb (15,10.00); Cm (37:39-4:1
4.83e3
148.1127
105.0707
132.0814
163.1233
252.1756
266.1546
288.1369
%
m/z

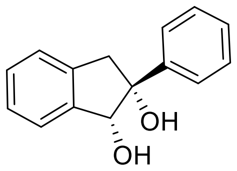

(1*R*, 2*R*)-2-phenyl-2,3-dihydro-1H-indene-1,2-diol, 22

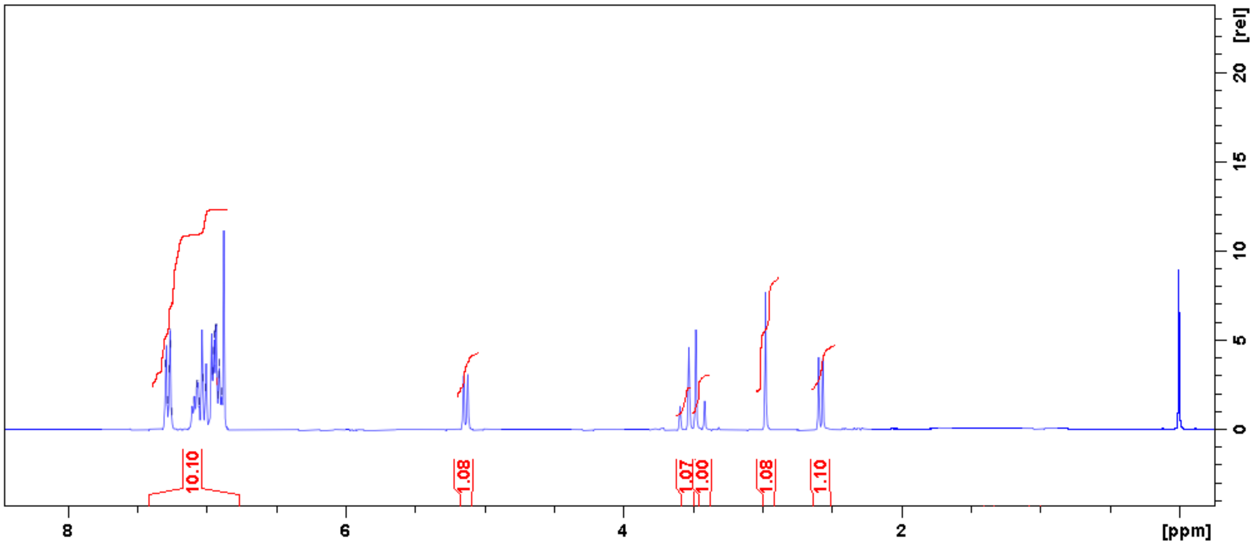

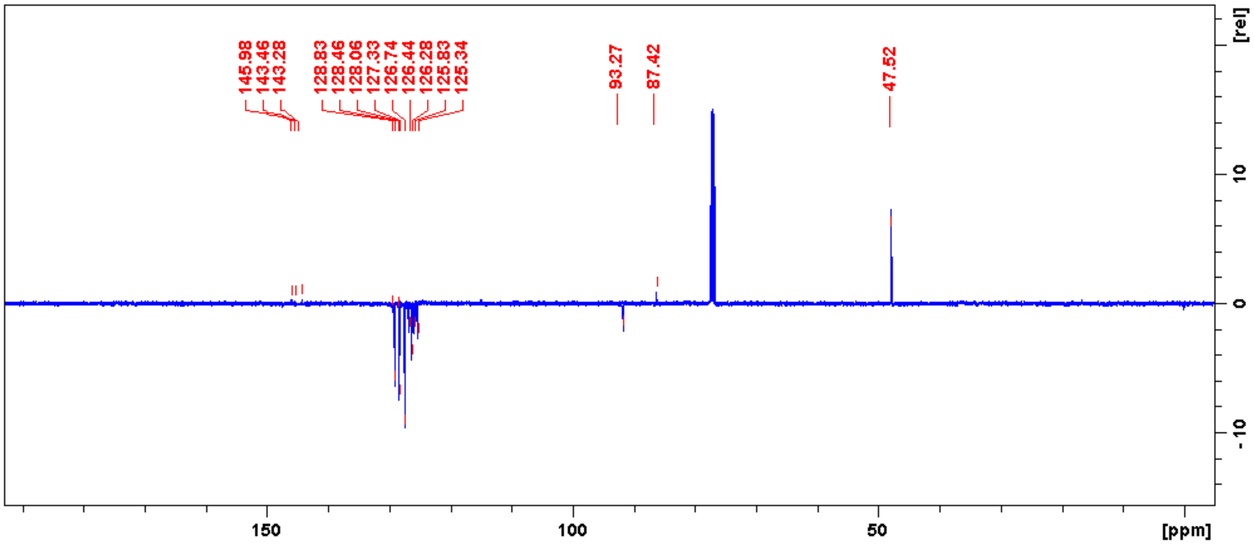

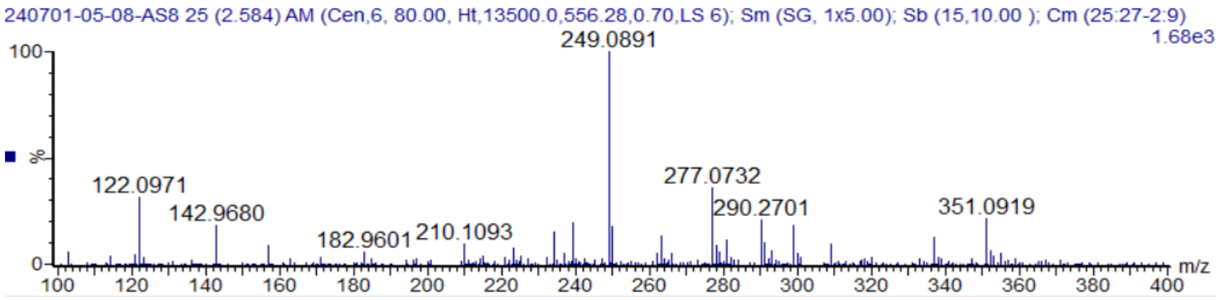

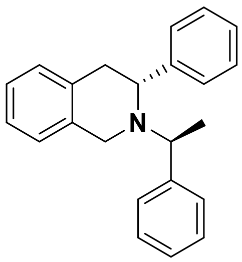

(*R*)-3-phenyl-2-((*S*)-1-phenylethyl)-1,2,3,4-tetrahydroisoquinoline, 23

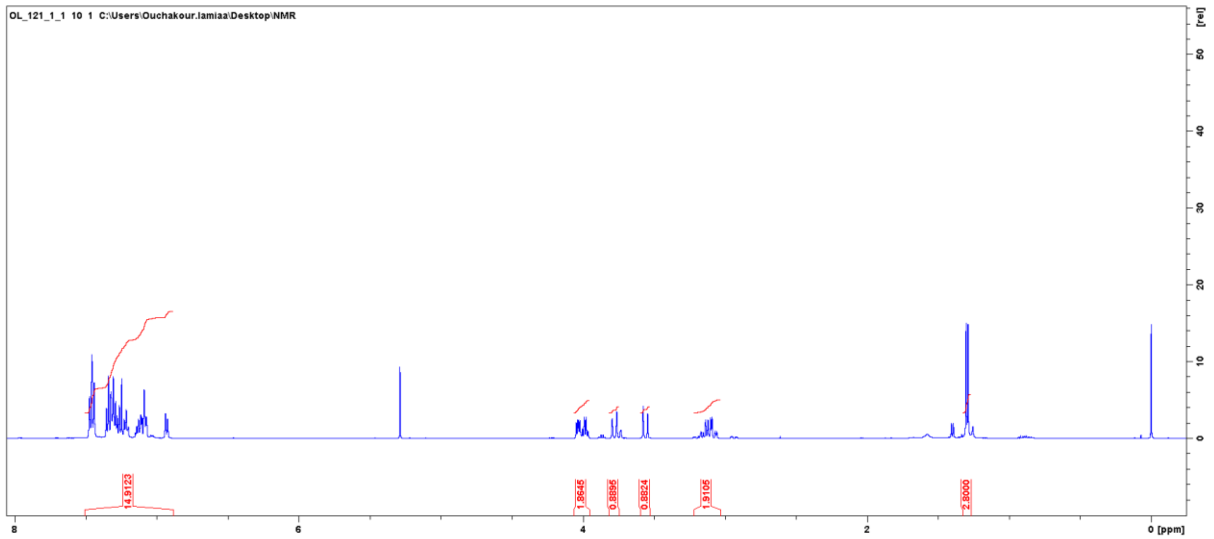

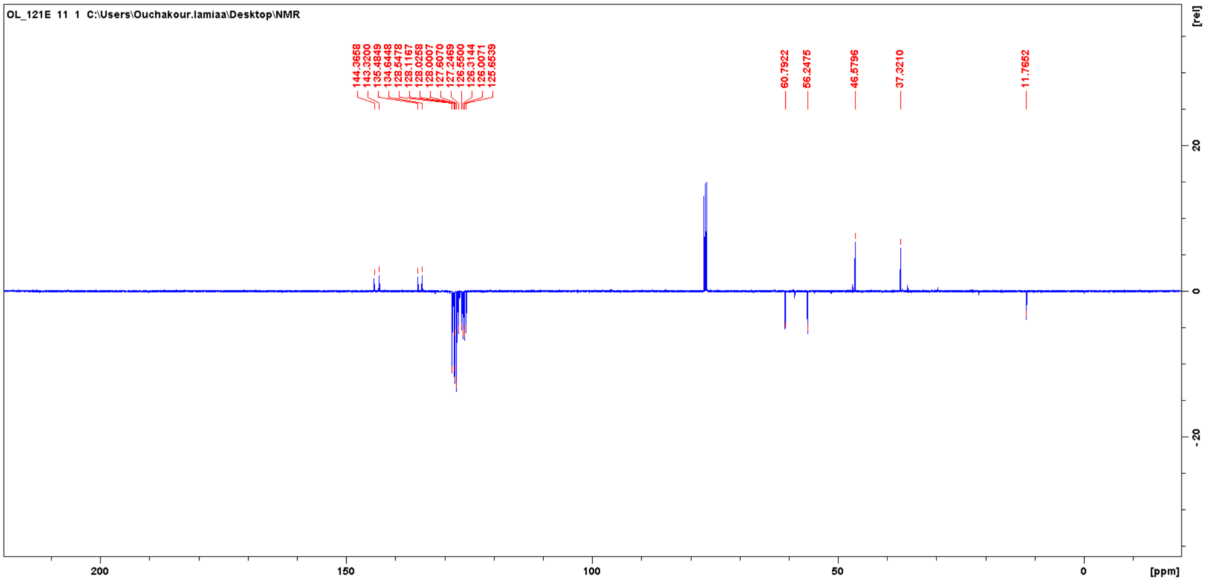

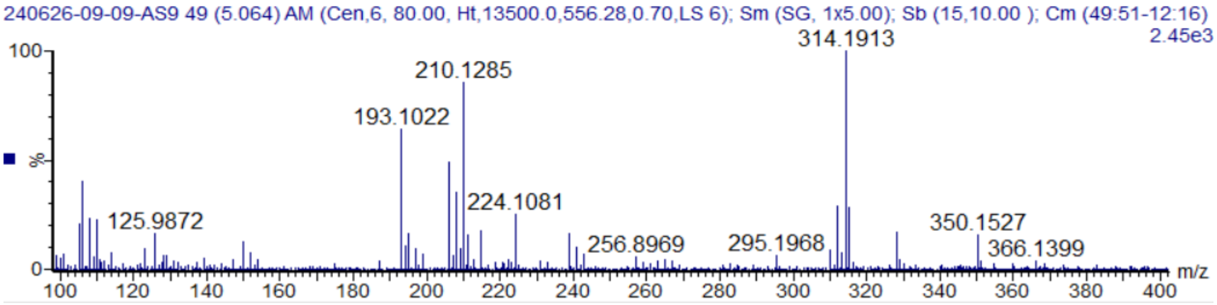

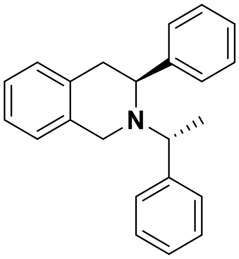

(*S*)-3-phenyl-2-((*R*)-1-phenylethyl)-1,2,3,4-tetrahydroisoquinoline, 24

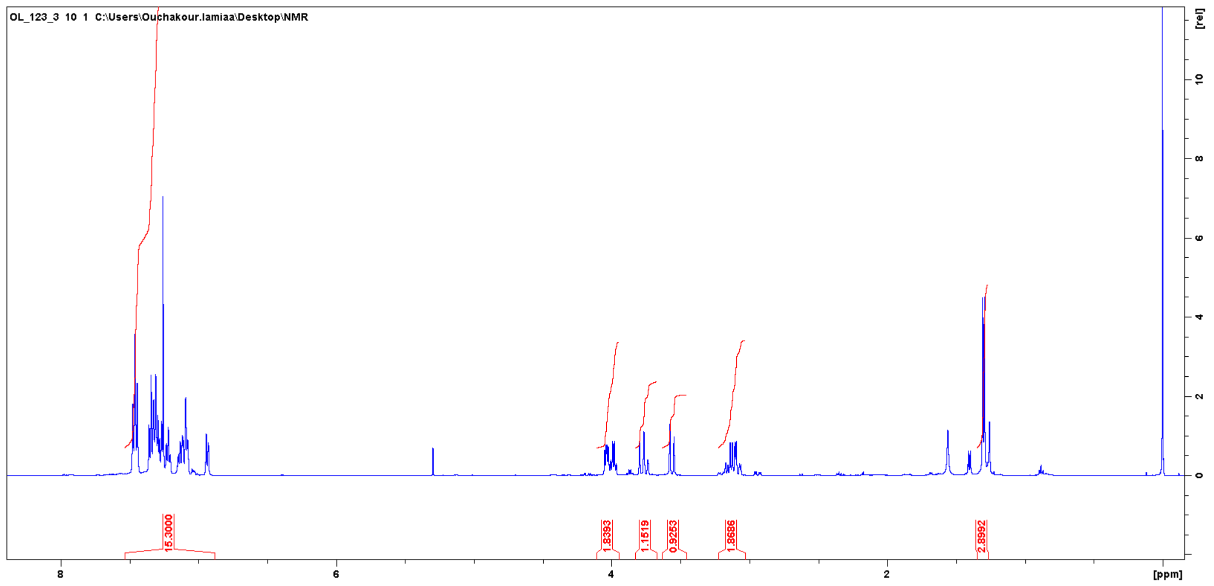
OL_123_3 10 1 C:\Users\Ouchakour.lamiaa\Desktop\NMR
15.3000
1.8393
1.1519
0.9253
1.8686
2.8992
[ppm]
[rel]

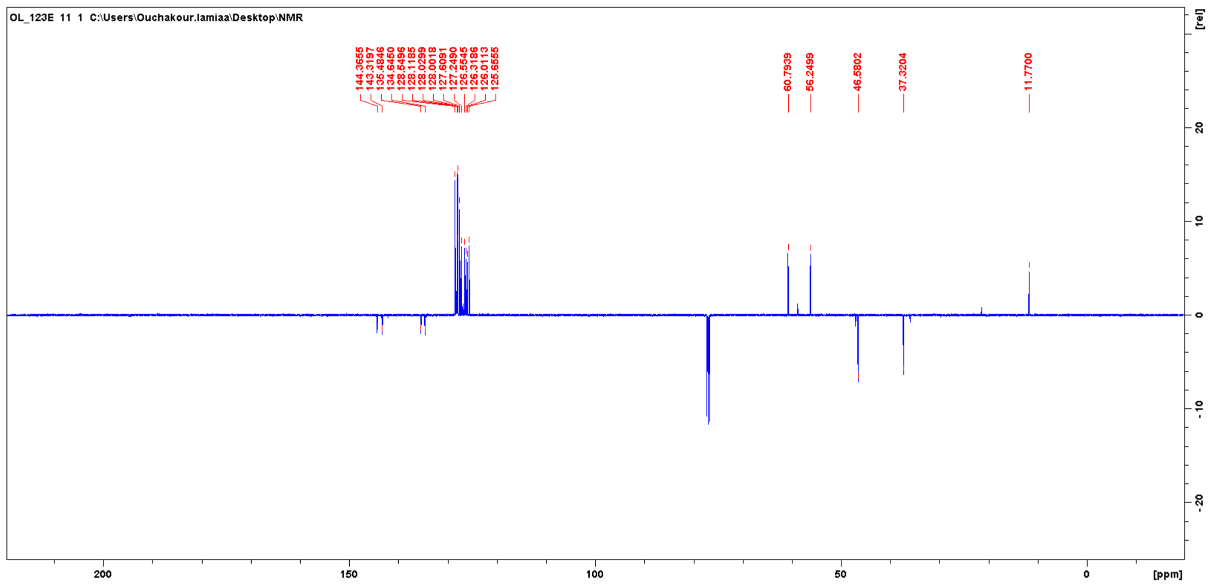
OL_123E 11 1 C:\Users\Ouchakour.lamiaa\Desktop\NMR
144.3655
143.3197
135.4846
134.6450
128.5496
128.1185
128.0299
128.0018
127.6091
127.2490
126.5545
126.3186
126.0113
125.6555
60.7939
56.2499
46.5802
37.3204
11.7700
[ppm]
[rel]

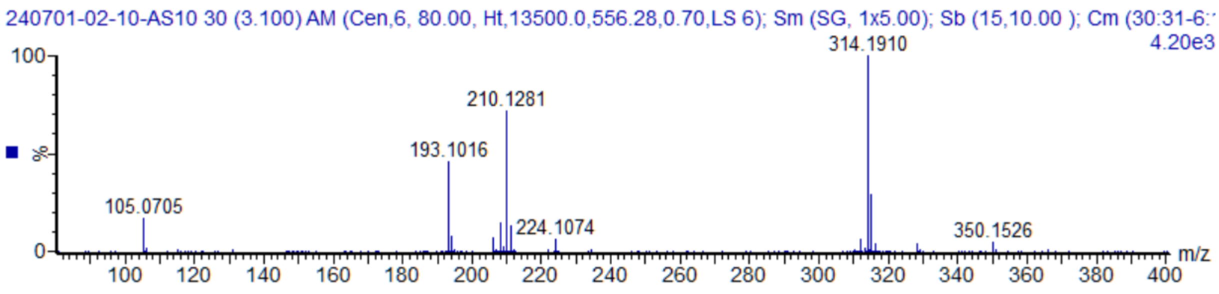
240701-02-10-AS10 30 (3.100) AM (Cen,6, 80.00, Ht,13500.0,556.28,0.70,LS 6); Sm (SG, 1x5.00); Sb (15,10.00); Cm (30:31-6:
4.20e3
314.1910
210.1281
193.1016
105.0705
224.1074
350.1526
%
m/z
